# Integrating plasma circulating protein-centered multi-omics to identify potential therapeutic targets for Parkinsonian cognitive disorders

**DOI:** 10.1186/s12967-025-06541-z

**Published:** 2025-05-12

**Authors:** Tayier Tuersong, Yu Xuan Yong, Yan Chen, Pei Shan Li, Samire Shataer, Munire Shataer, Liang Ying Ma, Xin Ling Yang

**Affiliations:** 1 Department of Pharmacy, Xinjiang Key Laboratory of Neurological Diseases, Xinjiang Clinical Research Center for Nervous System Diseases, Second Affiliated Hospital of Xinjiang Medical University, Ürümqi, 830001 Xinjiang People’s Republic of China; 2 Department of Neurology, Xinjiang Clinical Research Center for Nervous System Diseases, Xinjiang Key Laboratory of Neurological Diseases, Second Affiliated Hospital of Xinjiang Medical University, Ürümqi, 830001 Xinjiang People’s Republic of China; 3 Department of Histology and Embryology, Basic Medical College of Xinjiang Medical University, Ürümqi, 830001 Xinjiang People’s Republic of China

**Keywords:** Plasma circulating protein, Parkinson's disease, Mendelian randomization, Transcriptome analysis, Immune cells, Parkinson's disease dementia

## Abstract

**Background:**

Parkinson's disease (PD), the second most common neurodegenerative disease with notable clinical heterogeneity, has Parkinson disease dementia (PDD) that severely impacts patients' quality of life. As no effective treatment exists, this study aimed to find potential drug targets for PD cognitive disorders.

**Methods:**

Two-sample Mendelian randomization (MR) and transcriptome analysis were used to identify PD biomarkers. Protein-protein interaction (PPI), gene ontology (GO), and KEGG pathway analyses explored biological effects. A nomogram model was developed.

**Results:**

76 Mendelian randomization genes (MRGs) from MR and 1771 differentially expressed genes (DEGs) from the transcriptome were obtained. Three significant shared DEGs (S-DEGs) were identified, with USP8 and STXBP6 having strong diagnostic value for PDD. The nomogram model with these two genes showed enhanced predictive ability. These genes had physical interactions, co-localization, and correlated with ODC and NEU immune cells. USP8 was linked to five diseases, and STXBP6 to one.

**Conclusion:**

USP8, STXBP6, and immune cells (ODC and NEU) associated with PDD were identified, offering new insights into PD progression.

**Supplementary Information:**

The online version contains supplementary material available at 10.1186/s12967-025-06541-z.

## Introduction

Parkinson's disease (PD) is a neurodegenerative disorder characterized by motor symptoms such as tremors, rigidity, and bradykinesia [1]. However, the detrimental impact of non-motor symptoms, particularly cognitive impairment, on patients'quality of life has garnered increasing attention [2]. Epidemiological studies indicate that approximately 20–50% of PD patients exhibit mild cognitive impairment (MCI) in the early stages of the disease, and as it progresses, 20–40% will develop parkinson's disease dementia (PDD) [3–5]. PDD is marked by deficits in executive function, memory, and attention, significantly exacerbating functional dependence and caregiving burden. Currently, there are no effective therapies to reverse or delay its progression [6]. The classic pathological hallmark of PD is the degenerative loss of dopaminergic neurons in the midbrain substantia nigra, leading to reduced dopamine levels in the striatum and motor dysfunction [7]. However, the mechanism underlying cognitive impairment extends beyond a single neurotransmitter system disorder. Post-mortem studies have revealed widespread distribution of Lewy bodies (α-synuclein aggregates) in the brains of PDD patients, along with deterioration of the cholinergic system, activation of neuroinflammation, and impaired synaptic plasticity [8]. These multi-dimensional pathological changes suggest that cognitive impairment may result from multiple factors, including genetic susceptibility, protein homeostasis imbalance, immune disorders, and metabolic disturbances [9].

Despite rapid advancements in multi-omics techniques, existing studies have predominantly focused on single molecules or pathways, such as α-synuclein or mitochondrial dysfunction, failing to systematically integrate interactions at the multi-omics level [10]. Traditional studies often adopt"single omics"or"single marker"strategies, such as identifying risk genes through genome-wide association studies (GWAS) or screening differentially expressed proteins via proteomics [11, 12]. The high heterogeneity of PD makes it difficult for a single data level to fully capture the disease's nature. For instance, while GWAS-identified genetic risk loci are associated with PD onset, systematic evidence on their transcriptional regulation effects on protein function and subsequent cognitive decline is lacking [13]. Similarly, although proteomic studies have identified abnormal proteins in PD patient plasma or cerebrospinal fluid, the relationship between these proteins'dynamic changes and genetic background and immune status remains unclear [14]. Plasma proteins are considered ideal biomarker sources due to their non-invasive collection and dynamic reflection of physiological and pathological states. Studies show significant correlations between specific plasma protein levels and cognitive function scores in PD patients [15]. However, previous research has been limited to simple"protein-phenotype"associations, neglecting deeper exploration of how plasma proteins interact with genetic variation and transcriptional regulatory networks to influence PD cognitive phenotype. Are these proteins directly involved in key pathological processes like neuroinflammation and synaptic remodeling, or are they merely secondary phenomena? How can we distinguish"driving"targets from"accompanying"markers through multi-omics integration?

Moreover, the role of immune dysregulation in PD cognitive impairment has been underestimated. Recent evidence indicates that peripheral immune cells can infiltrate the central nervous system via the blood-brain barrier, releasing pro-inflammatory factors that exacerbate neurodegeneration [16, 17]. Multi-omics analysis of plasma proteins as mediators of immune-neural interaction could reveal the molecular basis of this process, but relevant research is still nascent [18]. To address these scientific challenges, this study integrates multiple omics data centered on plasma circulating proteins to systematically analyze the molecular network of cognitive impairment in PD. It aims to identify key interfaces between immune disorders and neurodegeneration and discover actionable therapeutic targets. Through Mendelian Randomization (MR), a causal association network of gene-transcript-protein was constructed, and cell type-specific regulation mechanisms were analyzed using single-cell transcriptome sequencing. The study explores how plasma protein-mediated peripheral immune disorders drive PD cognitive decline via neuroinflammation and synaptic dysfunction, filling a critical gap in the field. RNA sequencing verified candidate target expression and distribution in PDD patient brain tissues, providing a foundation for developing blood biomarkers and immunomodulatory therapies. In summary, this study places plasma circulating proteins at the center of the multi-omics network of PD cognitive impairment, revealing their role as a"molecular bridge"connecting genetic risk, immune disorders, and neurodegeneration. By addressing the two major scientific challenges of"multi-omics fault"and"immune mechanism deficiency,"this study aims to pave a new path for precision medicine in PD cognitive impairment.

## Methods

### Data sources

The single-cell dataset (E-MTAB-13437) included six adult bunched macaques, three of which exhibited stable PD-like symptoms, such as retardation, tetanus, and tremor, while the remaining three served as matched controls. Two datasets, GSE150696 and GSE20141, were downloaded from the GEO database (https://www.ncbi.nlm.nih.gov/geo/). GSE150696 was used as a validation set and consisted of whole blood samples from 12 PDD patients and 9 healthy controls. GSE20141, used as the training set, contained anterior cingulate cortex samples from 18 individuals, including 8 healthy controls and 10 PDD samples.

MR analysis utilized data from the GWAS database (https://gwas.mrcieu.ac.uk/), searching for outcome events and exposures using GWAS data. The IEUOpenGWAS database (https://gwas.mrcieu.ac.uk/) was accessed for whole blood or cerebrospinal fluid pQTL and genetic variation data related to PDD. The sample size consisted of 267 cases and 216,628 controls, with 1,6380,459 single nucleotide polymorphisms (SNPs), and the ethnic information was European.

### Mendelian randomization (MR) analysis

The harmonise_data function of the R package'TwoSampleMR'was used to align effect alleles and effect sizes. The MR function combined five algorithms (MR egger [19], weighted median [20], inverse variance weighted (IVW) [21], simple mode [22], and weighted mode [23]) to perform MR analysis. In this study, we primarily employed the inverse variance weighting (IVW) method to assess the causal relationship between exposure factors and outcomes. A *p*-value < 0.05 from the IVW method indicates a significant causal relationship between the two. The odds ratio (OR) was calculated, with values > 1 indicating a risk factor and values < 1 indicating a protective factor. Furthermore, sensitivity analyses were conducted to ensure the reliability of the results. These mainly included heterogeneity test, horizontal pleiotropy test, and leave-one-out analysis. The heterogeneity test was used to evaluate the degree of variation among different instrumental variables. If *p* > 0.05, it indicated that there was no significant heterogeneity among the instrumental variables. The horizontal pleiotropy test was utilized to detect whether the instrumental variables had other effects besides influencing the exposure factors and the outcomes. If *p* > 0.05, it suggested that there was no horizontal pleiotropy, meaning that the research results were not affected by confounding factors. The leave-one-out analysis involved removing each instrumental variable one by one and observing the impact on the results. If the results did not change significantly, it demonstrated that the analysis results were stable and reliable. Finally, we performed the MR-Steiger directionality test to determine whether the directionality of each exposure factor was correct, that is, whether they were consistent with the expected direction of the causal relationship.

### Functional enrichment analysis

To explore the biological functions and signaling pathways involved in differential genes, we used the R package'clusterProfiler'[24] for gene ontology (GO) and Kyoto Encyclopedia of Genes and Genomes (KEGG) enrichment analysis. GO analysis, based on the GO database, includes three categories: BP, cellular components (CC), and molecular functions (MF). KEGG pathway analysis annotates the pathways of identified proteins or differentially expressed proteins and analyzes key metabolic and signal transduction pathways involved in these proteins or genes.

### Analysis of differentially expressed genes (DEGs)

Differential expression analysis of PDD patients and healthy controls in the GSE20141 dataset was performed using the R package'limma'[25] and visualized with a volcano plot from the'ggplot2'[26] package. The threshold was set at |log2 FC| > 0.5, *p* < 0.05. The'VennDiagram'package in R was used to plot the intersection of transcriptome differential genes and Mendelian randomizetion differential genes, resulting in shared significant shared DEGs (S-DEGs).

### Identification of key genes

To evaluate the performance of key genes in disease diagnosis, receiver-operating characteristic (ROC) curves were plotted for several genes in both the training dataset (GSE20141) and the validation dataset (GSE150696). The classification ability of these genes was measured by the area under curve (AUC). Wilcoxon tests were used to analyze the expression levels of key genes with AUC > 0.6 in both datasets. Key genes with significant differential expression and consistent trends across both datasets were selected as biomarkers (*p < 0.05, **p < 0.01, ***p < 0.001).

The least absolute shrinkage and selection operator (LASSO) regression was performed using the'glmnet'[27] R package and 10-fold cross-validation was used to select candidate genes based on the optimal Log (Lambda) value. Next, an SVM-RFE model was constructed using the'e1071'R package to evaluate the importance of candidate genes and select the gene combination with the best performance based on error rate and accuracy. Venn diagram software was used to determine the final set of genes by intersecting the results from LASSO and SVM-RF.

### Key gene correlation analysis

In the training set (GSE20141), Spearman correlation analysis was performed between the key genes and all genes using the'psych'R package. Genes were ranked by their correlation values. Gene Set Enrichment Analysis (GSEA) pathway enrichment analysis was conducted using the'clusterProfiler'[24] R package, with the background gene set, to infer the signaling pathways potentially involving the key genes.

To explore immune cell differences between PDD samples and control samples, the CIBERSORT tool was used to estimate the proportion of immune cell types. Principal component analysis (PCA) clustering of immune cells was performed using the'ggplot2'package, and stacked histograms were plotted. Spearman correlation analysis was conducted to investigate immune cell interactions, and a correlation heatmap was generated with the'corrplot'package. Wilcoxon tests were used to analyze immune cell differences between the patient and control groups (*p* < 0.05), and violin plots were generated using the'ggplot2'package [26].

### Nomogram prediction model and GeneMANIA network construction

To evaluate the predictive efficacy of biomarkers for PDD, a nomogram was constructed based on biomarkers in the training set by utilizing the'RMS'package. The receiver operating characteristic (ROC) curve of the nomogram was plotted using the'pROC'package, and the AUC was calculated to assess the performance of the nomogram in predicting PDD. An AUC greater than 0.7 indicated that the prediction was accurate. The calibration curve was calculated using the'regplot'R package to verify the accuracy of the model. The decision curve analysis (DCA) was calculated using the'ggDCA'R package. The GeneMANIA network was built using the website https://genemania.org/ to explore relationships among key genes.

### Quality control (QC) of scRNA-seq data and cellular annotation

Before analyzing single-cell data, QC was performed. Gene expression in PDD patient tissue samples was explored at the single-cell level, and Seurat objects were created using the'Seurat'R package. QC criteria included 300–10,000 gene counts per cell and <10% mitochondrial gene expression. The FindMarkers function in Seurat was used to identify significant DEGs between cell types, with thresholds set to |log2 FC| ≥ 0.5 and *p* < 0.05. The VennDiagram package in R was used to visualize the intersection of these DEGs.

Global scaling normalisation (LogNormalize) was used to normalise the gene expression of each cell by the total expression, scalling by a factor of 10000. The'FindVariableFeatures'[28] function was used to filter the data, identifying the top 2000 highly variable genes after QC. UMAP was integrated with the RunMap function to classify the cellular taxa based on a dimensionality value of 40, with PCA plots generated for disease and control groups.The'FindAllMarkers'function was used to identify important marker genes in different clusters. The CellMarker website and the SingleR package were used to assist with cluster annotation.

### Identification of key cells

Single-cell data analysis was performed to study the expression level and distribution of key genes across different cell types in PDD and control groups.

### Cell–cell communication analysis and pseudo temporal analysis

Cell-cell communication analysis was performed using CellPhoneDB, which provides a database of ligands, receptors, and their interactions. Using CellChatDB.human as a reference, cell–cell interactions were explored for annotated cells. The R package Monocle 2 was used for pseudotemporal analysis of different subtypes within key cell clusters.

### Disease prediction

Key target genes were uploaded to the Metascape platform (https://metascape.org/gp/index.html#/main/step1) to explore diseases associated with key genes. The DisGeNET database was used to analyze significant disease association (*p* < 0.05), and the key gene-disease co-expression networks were visualized using NetworkAnalyst.

## Results

### MR analysis

**Fig. 1.**
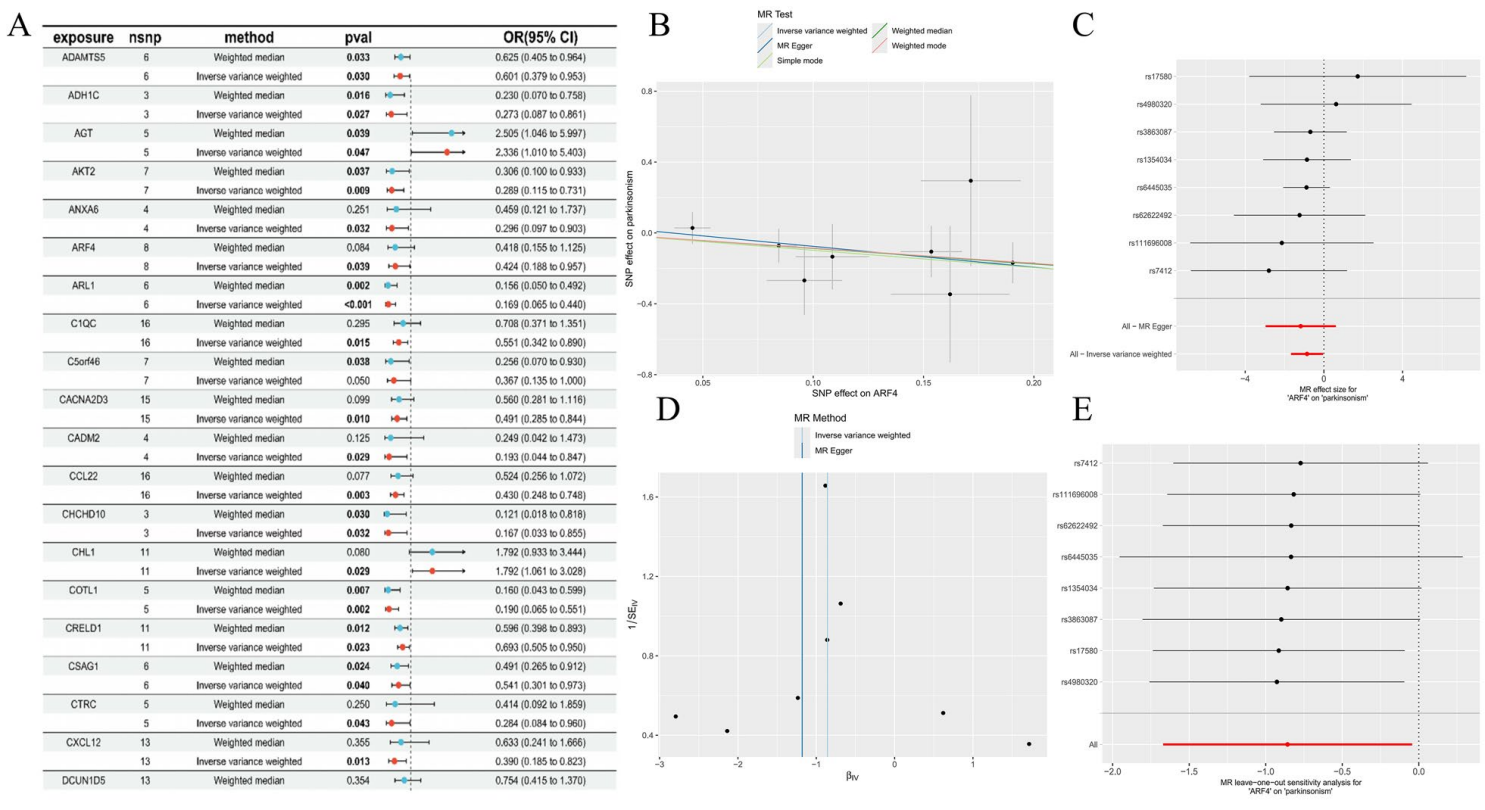
Statistical analysis of exposure effects using Mendelian randomization methods. **A** MR analysis results for different exposure variables, including gene loci, analysis methods, P-values, and odds ratios (only the first 20 were shown). **B** Shows the effect estimates of SNPs on outcome variables under various MR test methods. **C** Presents MR results of the associations between multiple SNP loci and diseases (such as Parkinsonism). **D** Compares the effect estimates of different MR methods. **E** The result of MR leave-one- out sensitivity analysis for the association between genes and outcome variables. Overall, through various graphical forms, it presents the results and method evaluations of MR analysis in gene–disease association research from different perspectives

To evaluate the effect of plasma circulating proteins on PDD, MR was performed, yielding 76 genes with significant causal associations with PD. A total of 56 were identified as safety factors and 20 as risk factors (Fig. 1A). Exposure factor-outcome correlation analysis identified ADAMTS5, ADH1 C, AGT, AKT2, ANXA6, ARF4, ARL1, C1QC, C5orf46, CACNA2D3, CADM2, CCL22, CHCHD10, COTL1, CRELD1, CSAG1, CTRC, CXCL12, DCUN1D5, DDAH1, etc. were safe factors (OR < 1), and AGT, CHL1, DDAH1, DHX8, were risk factors (OR > 1) (Fig. 1B). The estimated effects of the instrumental variable on the outcome corroborated the results of these analyses (Fig. 1C). Additionally, the randomness test confirmed that MR conformed to the random grouping of Mendel's second law (Fig. 1D). To further validate the MR analysis results, we performed sensitivity analyses, which included a heterogeneity test (Table S1), horizontal pleiotropy test (Table S2), leave-one-out analysis (Fig. 1E) and steiger directionality analysis (Table S3). These analyses confirmed that all 76 genes identified by MR were candidates with a significant causal relationship with PDD.

### Functional enrichment analysis

**Fig. 2.**
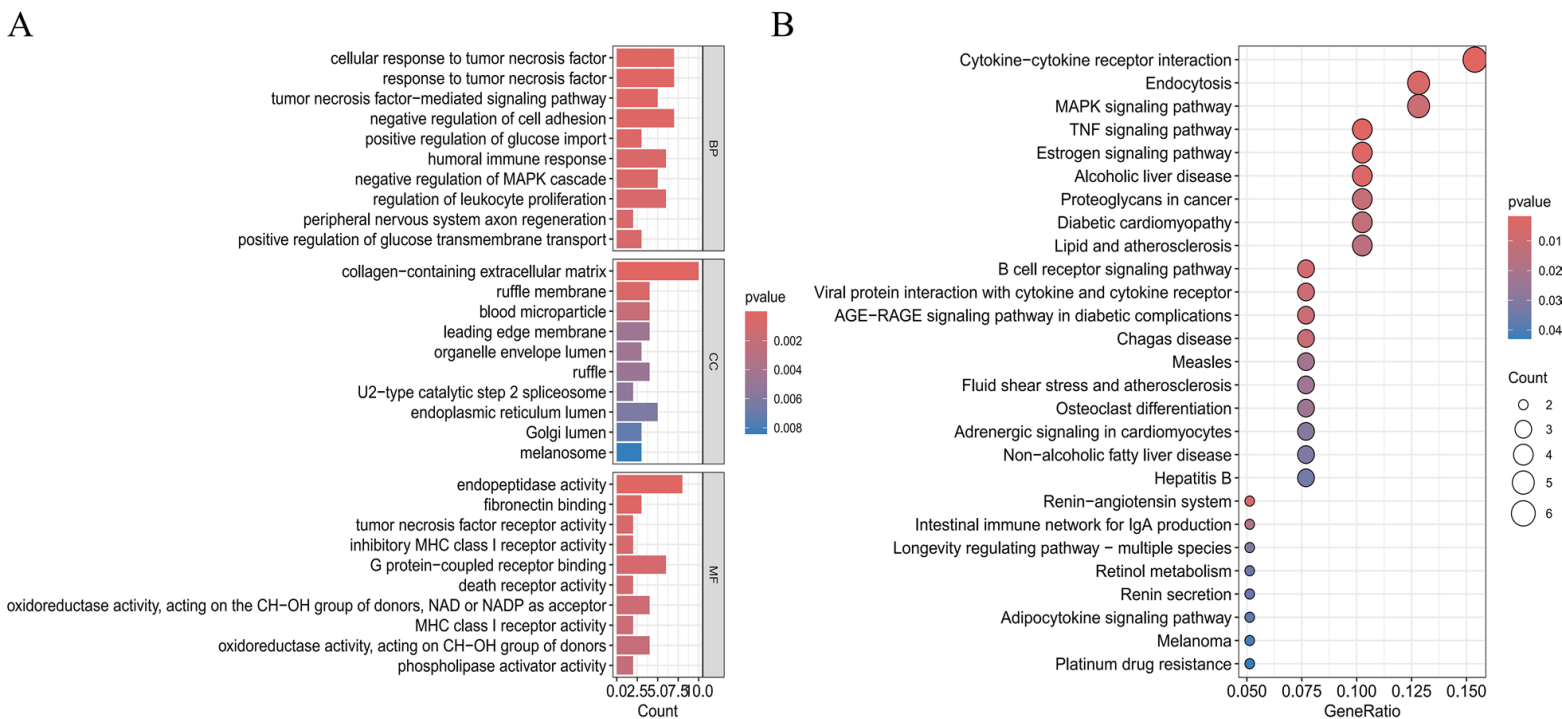
Functional enrichment analysis. **A **Gene Ontology (GO) enrichment analysis results. The bar length and color represent the count of enriched genes and the *p*-value, respectively. Shorter bars and more intense red colors indicate higher significance of enrichment for terms like"cellular response to tumor necrosis factor"and"collagen-containing extracellular matrix". **B** Kyoto Encyclopedia of Genes and Genomes (KEGG) pathway enrichment analysis results. Each dot represents a pathway, with the x-axis showing the GeneRatio, the y-axis listing pathway names such as"Cytokine - cytokine receptor interaction"and"MAPK signaling pathway". The dot color indicates the p-value and the size reflects the count of enriched genes

To explore the biological functions and signaling pathways with the differential genes, we performed GO and KEGG enrichment analyses. We identified 457 enriched GO biological functions, including 383 BP, 33 CC, and 41 MF. Additionally, 27 KEGG signaling pathways were significantly enriched. For BP, the significantly enriched functions were related to tumor necrosis factor-related responses, the negative regulation of cell adhesion, and the positive regulation of glucose uptake, highlighting the genes'roles in regulating metabolic responses and immune defense. In terms of CC, we observed significant enrichment in structures such as extracellular matrix, blood particles, and cytosolic membranes, suggesting these genes'involvement in cellular structure and functional component organization. The MF enrichment analysis revealed that these genes contributed to MF such as G protein-coupled receptor binding and fibronectin binding, emphasizing their importance in signaling and metabolic regulation (Fig. 2A). GO classifications with darker colors and larger positions in the outer ring (e.g., endopeptidase activity, fibronectin binding, etc.) indicated these functional terms were not only significantly enriched but may also represent key biological functions or molecular activities deserving further attention (Fig. 2B, S1 A). KEGG enrichment analysis revealed significant pathways related to immune regulation and inflammatory responses, including cytokine-cytokine receptor interaction, MAPK signaling, TNF signaling, and chemokine signaling. These findings suggest that the candidate genes may play pivotal roles in diseases such as PD, inflammation, atherosclerosis, and diabetic complications.

### Screening DEGs

**Fig. 3.**
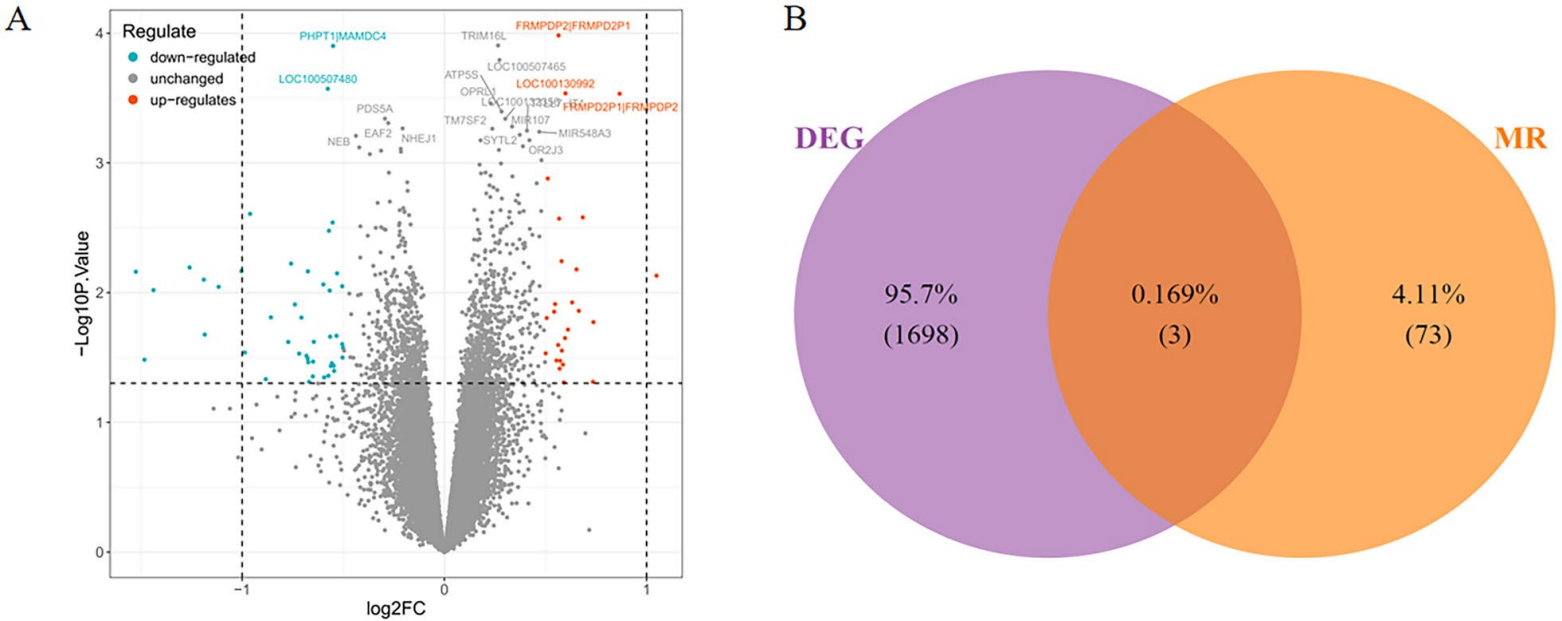
Screening of differentially expressed genes. **A** Volcano plot depicting gene expression changes. The x-axis represents the log2 fold-change (log2 FC), indicating the magnitude of gene expression alteration, and the y-axis shows the-log10 of the *p*-value, reflecting the statistical significance. Blue dots denote down-regulated genes, gray dots represent unchanged genes, and red dots indicate up- regulated genes. Some genes are labeled for identification. **B **Venn diagram illustrating the overlap between differentially expressed genes (DEG) and genes related to Mendelian randomization (MR)

Differential analysis revealed a total of 849 up-regulated and 852 down-regulated genes (Fig. 3A), with DEGs visualized in a heatmap (Fig. S1B). To identify genes that were differentially expressed in both the transcriptome dataset and MR analysis, we performed an intersection of transcriptome differential genes and MR differential genes, which identified three shared genes: USP8, STXBP6, and CTRC (Fig. 3B).

### Identification of key gene

**Fig. 4.**
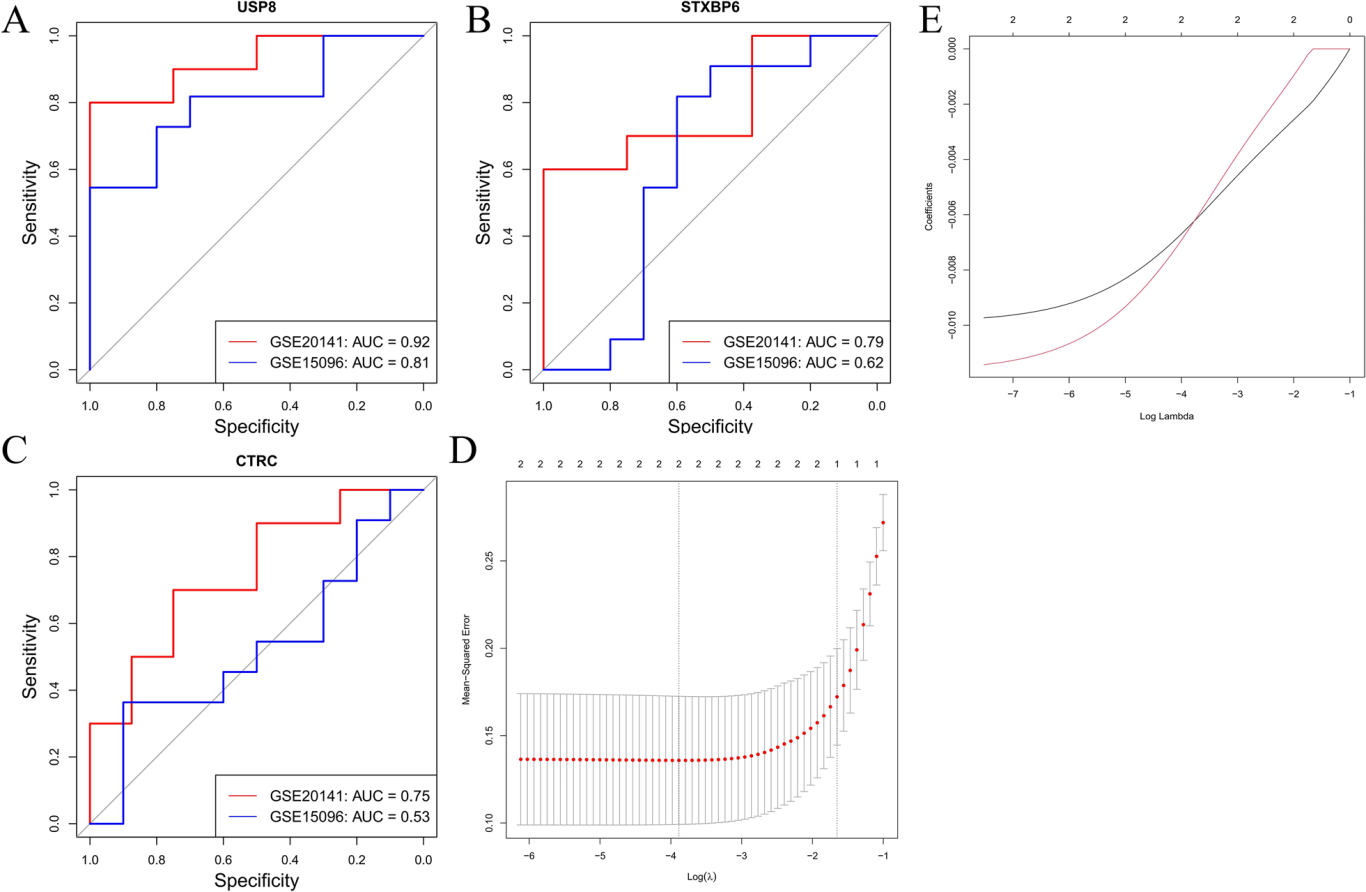
Gene-based diagnostic ROC curves and machine learning. **A–C** Receiver Operating Characteristic (ROC) curves for genes USP8, STXBP6, and CTRC respectively. Each plot compares two datasets (GSE20141 in red and GSE15096 in blue). The x-axis represents specificity, the y-axis represents sensitivity, and the area under the curve (AUC) values are indicated in the legend, showing the diagnostic performance of the genes in differentiating conditions. **D** Plot of mean - squared error vs. Log(λ) for selecting optimal λ in Lasso regression - based model; error bars show variability. **E** Plot of model coefficients vs. Log(λ) in Lasso regression analysis to visualize coefficient changes with regularization; two curves may represent different scenarios/variables

After identifying candidate genes, we evaluated the AUC values to measure their diagnostic capability in both training and validation datasets. In the GSE20141 training dataset, the AUC values for USP8 and STXBP6 were 0.92 and 0.79, respectively. In the GSE150696 validation dataset, the AUC values were 0.81 for USP8 and 0.62 for STXBP6. These results indicated that both USP8 and STXBP6 have good diagnostic potential in both datasets (Fig. 4A–C). Subsequently, we performed expression validation (Fig. S2), LASSO regression analysis and SVM-RFE (Fig. 4D, E, S3). The results showed that USP8 and STXBP6 genes exhibited consistent expression patterns across both datasets and displayed high stability and importance in both algorithms, supporting their potential as biomarkers.

### Key gene correlation analysis

**Fig. 5.**
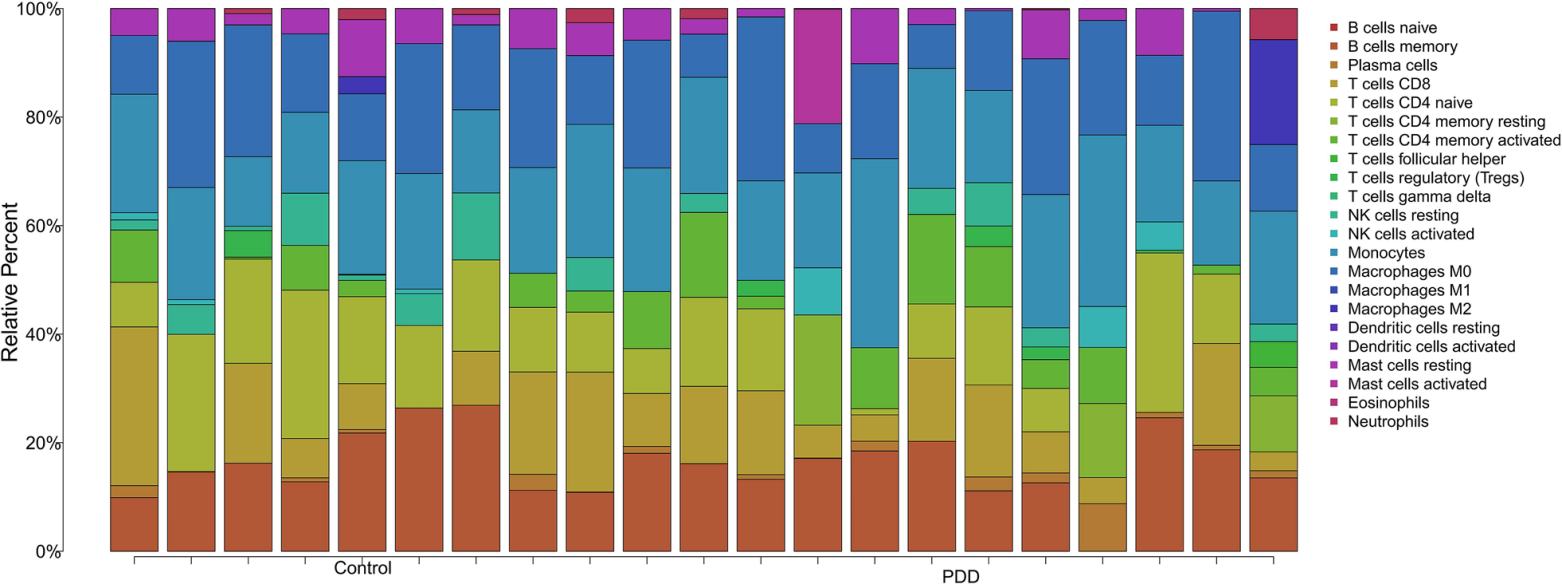
Immune infiltration analysis. Stacked bar chart vividly illustrates the relative percentage distribution of diverse immune cell types in the"Control"and"PDD"groups. Immune cell categories span from B cells (naive and memory) and T cells (including CD8, CD4 subsets, etc.) to NK cells, monocytes, macrophages (M0–M2), dendritic cells, mast cells, eosinophils, and neutrophils. It enables straightforward visual assessment of immune cell composition differences between the two groups, hinting at potential immunological shifts related to PDD

GSEA revealed that STXBP6 was enriched into the KEGG Medicus reference pathway related to electron transport complex I (Fig. S4). This pathway was influenced by mutations in SNCA, TDP43, and PINK1, which have all been implicated in PD.The immune profile of PDD patients exhibited unique patterns of immune cell composition, with notable changes in the proportion of specific cell types, such as NK cells, monocytes and macrophages M1, which may have critical pathological significance in PDD (Fig. 5, S5).

### Nomogram prediction model and GENEMANIA network construction

**Fig. 6.**
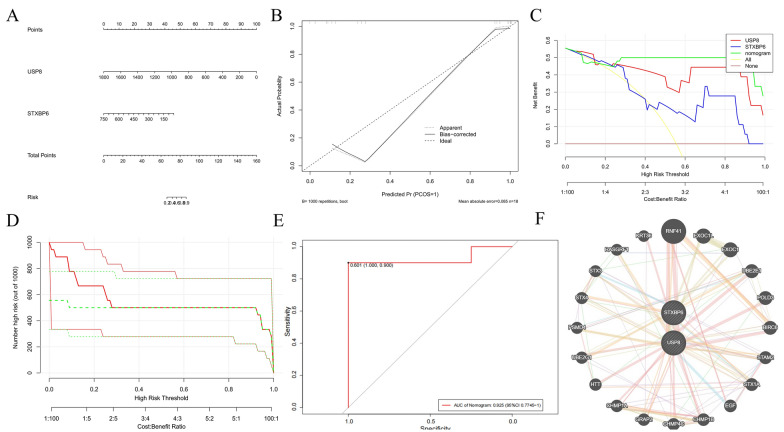
Analysis of key gene correlation. **A** Presents a visual tool for estimating AM risk by assigning points to biomarkers like USP8 and STXBP6. **B** Compares predicted and actual probabilities of AM occurrence. The dotted line is the ideal, while solid lines show apparent and bias - corrected predictions from 1000 repetitions. **C** Evaluates net benefit of different clinical strategies. Compares models (genes and nomogram) against treating all or none, helping set optimal risk thresholds. **D **Shows how cost-benefit ratios affect the number of high-risk cases out of 1000, highlighting practical clinical implications. **E** Assesses the nomogram's diagnostic performance for AM. With an AUC of 0.925 (95% CI 0.774–1), it plots sensitivity vs. specificity. **F** Displays interactions between key genes and related ones, revealing biological pathways and gene relationships linked to AM

The nomogram model based on USP8 and STXBP6 biomarkers demonstrated good predictive ability and clinical relevance for PDD risk prediction (Fig. 6A–D). The ROC model indicated that when the AUC of the nomogram is 0.925, the model has good accuracy (Fig. 6E). Additionally, the GENEMANIA network map showed that USP8 and STXBP6 genes have strong physical interactions and colocalization, suggesting they may be involved in a shared molecular complex or cellular process (Fig. 6F).

### QC of scRNA-seq data and Cell annotation

**Fig. 7.**
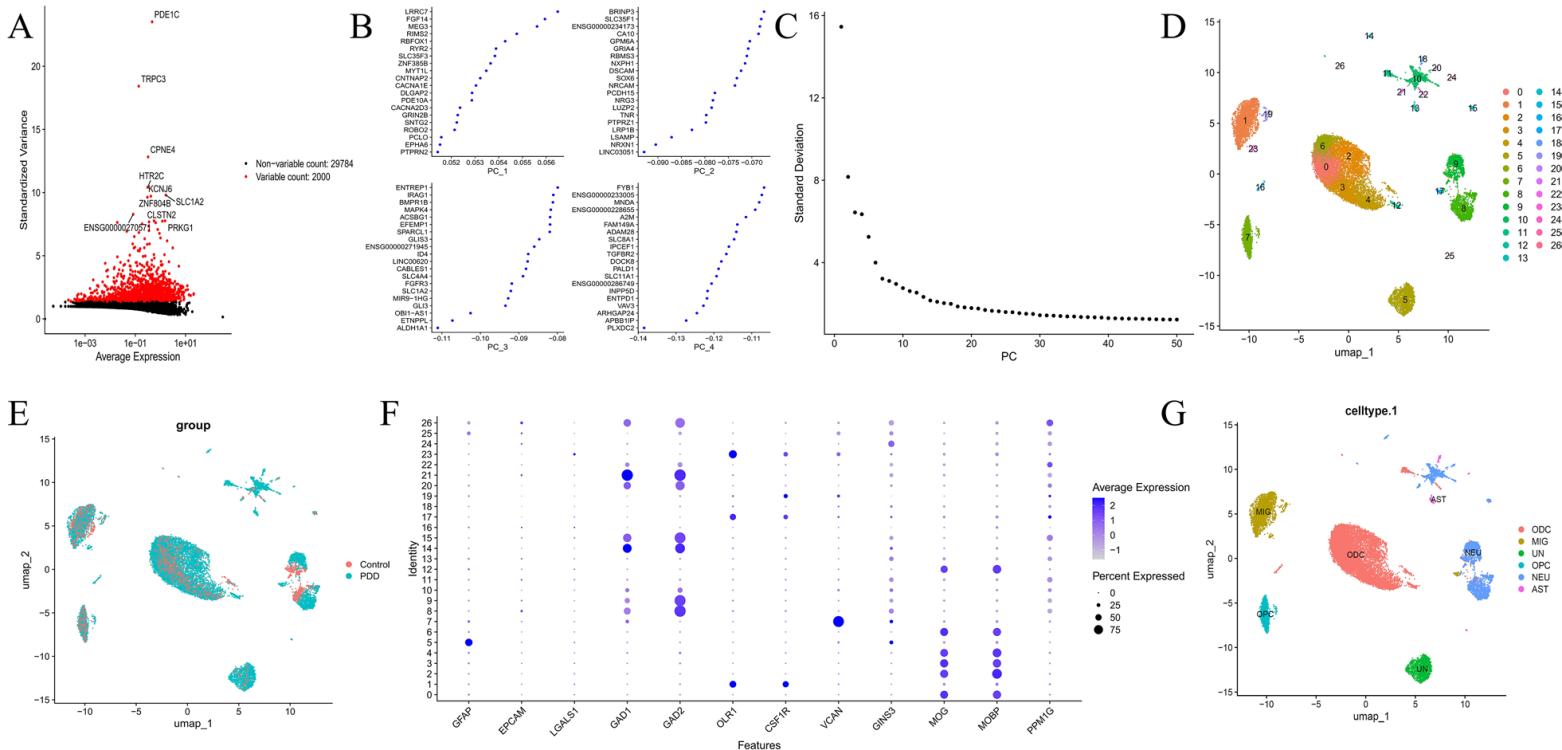
Multifaceted gene expression profiling: scatter, PCA, UMAP, and dot plot insights. **A** Scatter plot of average gene expression vs. standardized variance. Red dots: 2000 variable genes; black dots: 29,784 non-variable genes. Labeled genes like PDE1 C show notable variance - expression links. **B**, **C** In label (**B**), PCA plots (PC_1-PC_4) show gene-level data on principal axes, visualizing gene variance in reduced space. Label (**C**)’s line plot of standard deviation along PCs determines variance each PC captures, with the curve decreasing as variance explained decreases. **D**, **E** Label (**D**)’s UMAP plot has colored, clustered points, likely different cell types/samples with numbered group identities. Label (**E**)’s UMAP plot compares"Control"and"PDD"groups, using color to clearly show differences in gene expression patterns between them. **E **Dot plot shows gene “Identity” by average expression (color) and expression percentage (dot size). **G** UMAP plot colors points by cell types (e.g., ODC, MG), visualizing cell type distribution by gene expression

In the single-cell transcriptomic analysis of PDD-associated data, we first performed rigorous data QC. This resulted in 31,784 genes and 18,340 cells being retained for further analysis (Fig. S6 A, B). After QC, the top 2000 highly variable genes were identified (Fig. 7A), including PDE1 C, TRPC3, CPNE4, etc. Linear dimensionality reduction was applied, identifying an optimal dimensionality value of 40 for cell clustering, with UMAP and PCA maps generated to classify cell clusters, providing insights into disease and control group cell types (Fig. 7B–E). We annotated six distinct cell types: ODC, MIG, UN, OPC, NEU, and AST (Fig. 7F, G).

### Identification of key cells

**Fig. 8.**
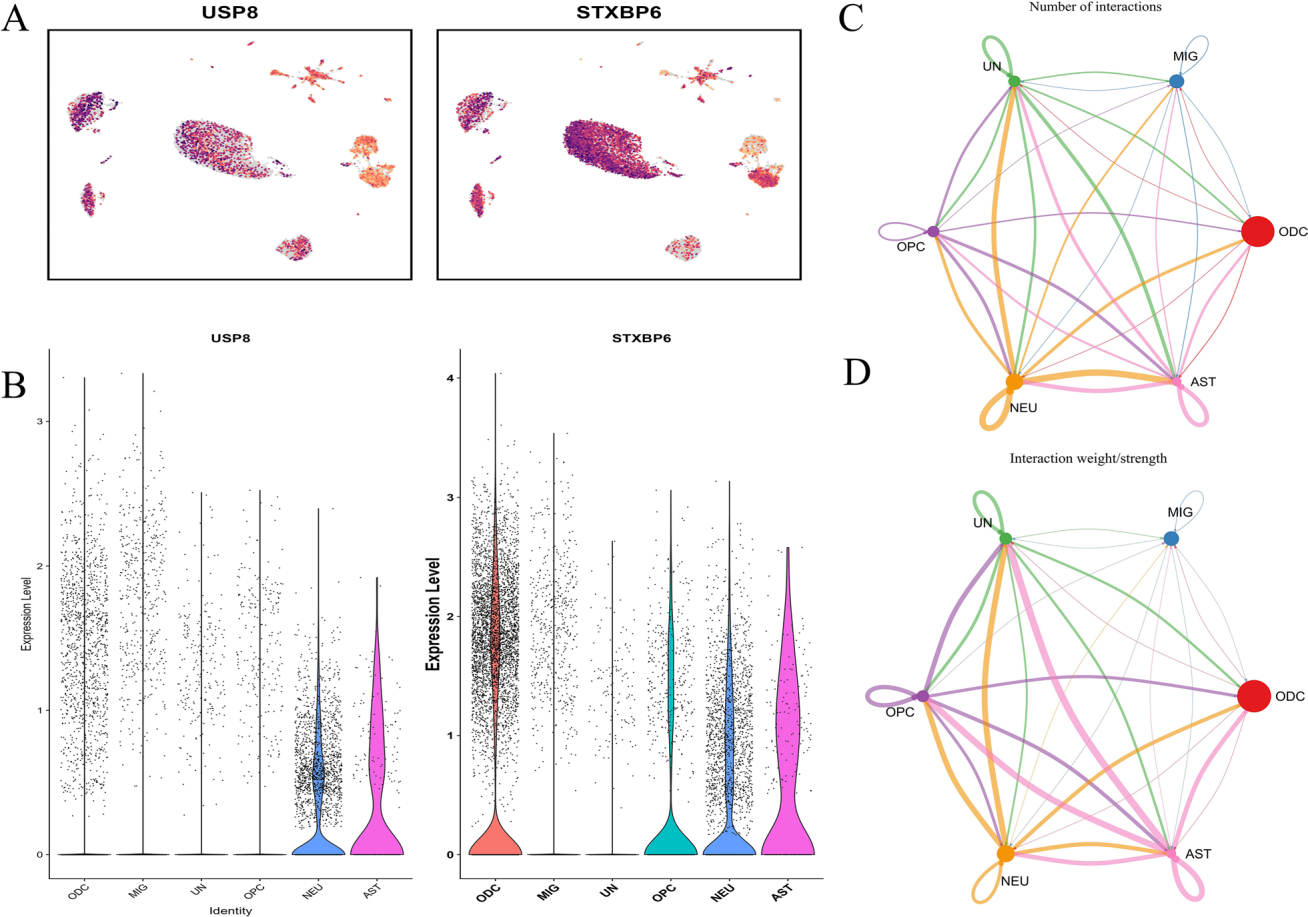
Gene Expression Profiles of USP8 & STXBP6 and Cellular Communication Dynamics Depicted in Interaction Maps. **A** UMAP plots for USP8 and STXBP6 genes. They display gene expression across cell identities, with colored clusters for different cell types, pinpointing where these genes are expressed. **B** Violin plots of USP8 and STXBP6 expression across cell identities like ODC and MIG. Width shows data density at different levels, comparing expression distributions. **C**, **D** Cellular communication interactions maps. Label (**C**) shows the number of interactions among cell identities, with nodes as cell identities and connecting lines indicating interactions, where line-related features suggest frequency or strength. Label (**D**) focuses on the interaction weight/strength among these cell identities, providing a detailed look at connection strengths to aid understanding of cell–cell communication relationships

By analyzing single-cell data from the PDD and control group (Fig. 8A, B), we investigated the expression level and distribution of USP8 and STXBP6 across different cell types. Notably, both genes showed high expression in specific cell types, particularly AST cells, with significant expression also observed in NEU cells. This suggests that AST and NEU cells may play a crucial role in the pathogenesis of PDD.

### Cell-cell communication analysis

To investigate cell–cell interactions in the annotated single-cell dataset, we performed cell communication analysis (Fig. 8C, D, S7). The results indicated that ODC cells were central in the network, exhibiting the highest number and intensity of interactions, particularly with NEU and AST cells. This suggests that ODC cells play a key role in maintaining homeostasis, myelination, and neural network function. NEU cells, as the primary functional cells, had strong interactions with AST and ODC, further supporting their role in signaling and functional regulation of the nervous system.

### Pseudotime analysis of the key cells

Pseudotime trajectory analysis of the key cell clusters ODC (Fig. S8) and NEU (Fig. S9) revealed distinct developmental trajectories between the PDD and control groups. Although there was a similarity in the pseudotemporal distribution between the two, the density or aggregation of the cell clusters differed in different states. The PDD group showed more aggregation of cells along a certain branch, which may imply that in the disease state, the cells were more inclined to move towards a particular developmental or differentiation direction, suggesting that the disease state may have affected the normal developmental path of the cells. Pseudotime analysis allowed us to see the differentiation trajectory of NEU cells in different states. Cells under control and PDD conditions showed certain differences in development, especially in specific states and branches, there may be more cell aggregation, suggesting that the disease state may affect the normal differentiation process of cells. The expression of STXBP6 and USP8 genes fluctuated significantly during the pseudotime course, so it played an important role in the development of cells.

### Disease prediction

Visualization of the key target gene-disease co-expression network on the NetworkAnalyst platform revealed six diseases associated with the key genes (Fig. S10). USP8 may be closely associated with a variety of diseases, including pituitary adrenocorticotropic hormone-secreting adenomas, pituitary-dependent Cushing's disease, esophageal squamous cell carcinoma, hereditary spastic paraplegia, and autosomal recessive spastic paraplegia type 59. In addition, USP8 was involved in the ubiquitin-proteasome degradation pathway, which was important for the degradation of key proteins in neurodegenerative disorders such as PD. STXBP6 (associated with childhood autism) may modulate synaptic function, and synaptic dysfunction plays an important role in neurodegenerative processes in PD.

## Discussion

This multi-omics study integrates MR, transcriptomics, immune infiltration analysis, and single-cell profiling to elucidate the molecular mechanisms underlying PDD. Our results pinpoint key causal genes, reveal immune-metabolic interactions, and delineate cellular dysfunction at single-cell resolution, thereby providing new insights into PDD pathogenesis and potential therapeutic avenues. In the following section, we situate these findings within the broader context of neurodegenerative disease research, emphasizing consistencies, discrepancies, and conceptual progress.The MR analysis identified 76 plasma proteins with causal links to PDD, including protective (ADAMTS5, ADH1 C) and risk (AGT, CHL1) factors. These genes converge on pathways critical to neurodegeneration, such as extracellular matrix remodeling, retinoid signaling, and synaptic plasticity. For instance, ADAMTS5, a metalloprotease previously linked to osteoarthritis, emerged as a neuroprotective factor. Its role in cleaving versican, a pro-inflammatory proteoglycan, may mitigate neuroinflammation by reducing inflammatory substrate accumulation in the brain parenchyma [29]. This aligns with recent proteomic studies implicating matrix metalloproteases in PD progression but expands their relevance to cognitive decline [30]. Similarly, ADH1 C’s involvement in retinol metabolism underscores the underappreciated role of retinoid signaling in dopaminergic neuron survival—a pathway corroborated by preclinical models showing retinoic acid deficiency exacerbates hippocampal atrophy [31].

The risk gene AGT, a key component of the renin-angiotensin system (RAS), highlights the intersection of systemic hypertension and neurodegeneration. Elevated AGT may exacerbate blood-brain barrier (BBB) dysfunction, facilitating neurovascular injury via angiotensin II-driven microglial activation [32]. This mirrors findings in Alzheimer’s disease, where RAS hyperactivity amplifies amyloid-β toxicity, suggesting shared mechanisms across dementias [33]. However, our reliance on European GWAS data limits generalizability, as PD-associated loci like GBA exhibit ancestry-specific frequencies [29]. Future studies must prioritize diverse cohorts to disentangle genetic and environmental interactions, particularly for ADH1 C, where alcohol consumption may modulate its activity in PDD progression.In a comparable study, a multi-omics investigation utilizing metabolomics and metagenomics techniques in a cynomolgus monkey model revealed an association between gut microbial metabolites, such as PDPC, and a Parkinson's disease-associated gene (SLC5 A3). However, this study did not conduct an in-depth analysis of immune cell phenotypes, thereby limiting the exploration of underlying immune mechanisms [34, 35]. Although the meta-analysis of genetic variation integrated multi-lineage GWAS data and identified 78 risk loci, the absence of functional omics validation, such as proteomics, limits the in-depth analysis of the specific functions and mechanisms of these genetic variants in disease pathogenesis [29]. A comprehensive comparison reveals that this study employs a more systematic and innovative methodological approach, particularly in examining the linkage between peripheral immunity and central nervous system pathology.

The intersection of MR-derived genes and transcriptomic differentially expressed genes (DEGs) pinpointed USP8 and STXBP6 as central to PDD pathogenesis. Both genes demonstrated robust diagnostic utility, outperforming existing biomarkers [36]. USP8, a deubiquitinase regulating endosomal sorting (ESCRT), is critical for α-synuclein clearance. Its downregulation in PDD likely destabilizes lysosomal membranes, impairing proteostasis and exacerbating Lewy body pathology—a mechanism supported by studies linking USP8 mutations to pituitary adenomas via secretory vesicle dysregulation [37]. Conversely, STXBP6, a syntaxin-binding protein, governs synaptic vesicle docking and mitochondrial electron transport. STXBP6 knockdown in iPSC-derived dopaminergic neurons recapitulates PD-associated metabolic stress, reducing mitochondrial membrane potential and elevating ROS [38]. This dual role in neurotransmission and bioenergetics positions STXBP6 at the nexus of synaptic and mitochondrial dysfunction, a hallmark of PD progression.These findings extend prior work on ubiquitin-proteasome and SNARE complex dysfunction in neurodegeneration. For example, USP8’s interaction with ESCRT-III parallels Parkin-mediated mitophagy in PD, while STXBP6’s homology to Munc18-1, a protein mutated in early-onset PD, suggests evolutionary conservation of synaptic maintenance mechanisms [39]. The diagnostic superiority of combining USP8 and STXBP6 further underscores the value of multi-omics integration in resolving heterogeneous pathologies like PDD.In this study, both USP8 and STXBP6 have strong diagnostic potential for distinguishing PDD patients from healthy controls, making them promising biomarkers for early diagnosis or disease assessment.

Functional enrichment analyses revealed immune-inflammatory (TNF/NF-κB, cytokine-cytokine receptor) and metabolic (oxidative phosphorylation) pathways as central to PDD. Elevated TNF-α and IL-6 levels correlate with microglial activation and BBB breakdown, facilitating monocyte infiltration and perpetuating neuroinflammation [40]. Monocyte-derived macrophages adopting an M1 phenotype release IL-1β, which activates astrocytes to produce complement proteins (e.g., C3), driving excessive synaptic pruning [41]. This aligns with proteomic studies identifying plasma proteins like AGT as molecular bridges between peripheral immunity and central neurodegeneration [18]. Mitochondrial dysfunction emerged as another pillar of PDD pathogenesis. STXBP6’s association with Complex I deficiency mirrors PINK1-linked mitophagy defects, suggesting convergent pathways in sporadic and familial PD [42]. Dysfunctional mitochondria accumulate in PD neurons, generating ROS that oxidize dopamine to toxic quinones—a process amplified by STXBP6 downregulation. These results reinforce the “multiple-hit” hypothesis of PD, where genetic, environmental, and metabolic insults synergize to trigger neurodegeneration.Immunoinfiltration analysis revealed significant differences in immune cell populations between PDD patients and control samples. PCA demonstrated distinct distributions and correlations of immune cells within these groups. For instance, macrophage and dendritic cell activity may be altered in PDD patients, with aberrant macrophage activation exacerbating neuroinflammation [43]. Similarly, dysfunctional dendritic cells may impair immune regulation and destabilize the neuronal microenvironment, contributing to PDD progression [44]. These findings highlight the importance of immunological abnormalities in PDD pathogenesis and provide a foundation for developing immunomodulatory treatments [45].

Single-cell clustering implicated oligodendrocytes (ODCs) and neutrophils (NEUs) in PDD pathophysiology. ODC maturation arrest correlates with hypomyelination and axonal degeneration, a phenomenon observed in postmortem PD brains [46]. Conversely, NEUs exhibit accelerated maturation and oxidative burst activity, driven by STXBP6-mediated metabolic reprogramming. These cells secrete myeloperoxidase (MPO), generating hypochlorous acid that exacerbates neuronal loss—a mechanism previously unrecognized in PD.Cell-cell communication analysis highlighted disrupted ODC-NEU interactions via semaphorin-plexin signaling. Semaphorin 3 A, released by stressed ODCs, binds neuronal plexin A4, inducing axonal retraction [47]. This pathway, critical during development, may be co-opted in PDD to drive circuitopathy. Notably, the expression patterns of astrocytic USP8 and neuronal STXBP6 indicate cell-type-specific functions. Specifically, USP8 regulates NF-κB-dependent cytokine release in astrocytes, whereas STXBP6 precisely modulates synaptic vesicle recycling in neurons.Cell communication analyses showed that receptor-ligand interactions between different cell types may be altered in PDD. Abnormal communication between ODC cells and other cells may disrupt myelination processes, affecting the speed and accuracy of nerve signal transmission [48]. Pseudotemporal trajectory analysis revealed differential aggregation patterns of key ODC and NEU cells between PDD and control groups. The expression of STXBP6 and USP8 fluctuated during the pseudotime course, suggesting their involvement in the developmental trajectories of these cells and their contribution to PDD pathogenesis. Finally, disease-gene co-expression network analysis identified six diseases significantly associated with USP8 and STXBP6. USP8 is linked to a variety of diseases, including pituitary adenomas, Cushing's disease, and esophageal squamous cell carcinoma. STXBP6 is associated with childhood autism, and its role in synaptic dysfunction may contribute to neurodegeneration in PD. These findings suggest that these genes may play indirect roles in PD pathology, highlighting the need for further research to validate these associations and explore potential therapeutic targets.

Despite the comprehensive exploration of molecular mechanisms underlying PDD through an integrated multi-omics approach, and the identification of potential biomarkers and therapeutic targets, several limitations persist. Firstly, the single-cell transcriptome analysis, conducted on a limited sample size of six adult macaques (three with PD-like symptoms and three controls), may not fully capture the cellular heterogeneity observed in human PDD patients. Secondly, differences in neurodegenerative disease pathologies between the macaque model and humans could limit the direct translational value of the findings. Thirdly, MR analyses primarily utilized genetic data from European populations (GWAS database), which may affect the generalizability of results due to genetic background variations across races. Although key genes (e.g., USP8, STXBP6) and their associations with PDD were identified via MR and transcriptome analysis, the specific functional mechanisms of these proteins have not been validated through in vitro or in vivo experiments. Sensitivity analysis confirmed the robustness of the results; however, undetected confounding factors may still exist. Immunoinfiltration analysis revealed changes in the proportions of specific immune cells (e.g., NK cells, monocytes) in PDD patients but did not further investigate the activation status or functional phenotypes of these cells. Diagnostic models, such as nomograms based on USP8 and STXBP6, were evaluated only within the training set (GSE20141) and validation set (GSE150696). The limited sample size and lack of independent external cohorts might lead to an overestimation of the clinical efficacy of these models. Additionally, the study predominantly relied on transcriptome and genetic data without directly measuring the expression levels of candidate proteins (such as USP8 and STXBP6) in plasma or brain tissue of PDD patients, thus failing to confirm the consistency between transcription and protein expression. Lastly, given the high heterogeneity in clinical manifestations and pathological progression of PDD, the study did not stratify different subtypes (e.g., rapid vs. slow progression) based on molecular characteristics.Future work will involve the utilization of human iPSC-derived neurons and brain tissue to validate the underlying mechanism. Additionally, extended single-cell analysis will be conducted within the human PDD cohort. Multi-lineage GWAS integration will be performed to enhance universality. The functional impact of the protein will be confirmed through rigorous experimental validation. Furthermore, flow or spatial transcriptome analysis will be employed to investigate immune activation. External validation of the diagnostic model will also be carried out. Finally, precision therapy will be guided by molecular typing.

## Conclusion

This study identifies USP8 and STXBP6 as multi-omics hubs driving PDD through proteostasis, synaptic integrity, and mitochondrial dysfunction. Their diagnostic potential is validated by machine learning models, and their cross-disease links (e.g., autism, pituitary adenomas) reveal shared molecular pathways. Single-cell analyses highlight oligodendrocytes and neurons as key players, with disrupted ODC–NEU interactions exacerbating cognitive decline. By integrating genetic, immune, and cellular insights, this work provides a roadmap for targeted therapies, advancing PDD research toward mechanism-based interventions and personalized treatment strategies.

## Supplementary Information

Additional file 1.

## Data Availability

All data generated and analyzed during this study are included in this article.

## Abbreviations

PD: Parkinson's disease
MR: Mendelian randomization
PPI: Protein-protein interaction
GO: Gene ontology
KEGG: Kyoto Encyclopedia of Genes and Genomes
DEGs: Differentially expressed genes
S-DEGs: Significant shared DEGs
PDD: Parkinson's disease dementia
MCI: Mild cognitive impairment
SNPs: Single nucleotide polymorphisms
ROC: Receiver-operating characteristic
AUC: Area under curve
LASSO: Least absolute shrinkage and selection operator
SVM-RFE: Support vector machine - recursive feature elimination
GSEA: Gene Set Enrichment Analysis
PCA: Principal component analysis
scRNA seq: Single-cell RNA sequencing
QC: Quality control
UMAP: Uniform manifold approximation and projection
BP: Biological processes
CC: Cellular components
MF: Molecular functions
OR: Odds ratio
GWAS: Genome-wide association studies
