# Supplementary material for: Integrating plasma circulating protein-centered multi-omics to identify potential therapeutic targets for Parkinsonian cognitive disorders
Source: J Transl Med. 2025 May 12;23:535. doi: 10.1186/s12967-025-06541-z (PMC12070786; doi:10.1186/s12967-025-06541-z)
Supplement: Supplementary file 1 — Additional file 1. [file 12967_2025_6541_MOESM1_ESM.doc]

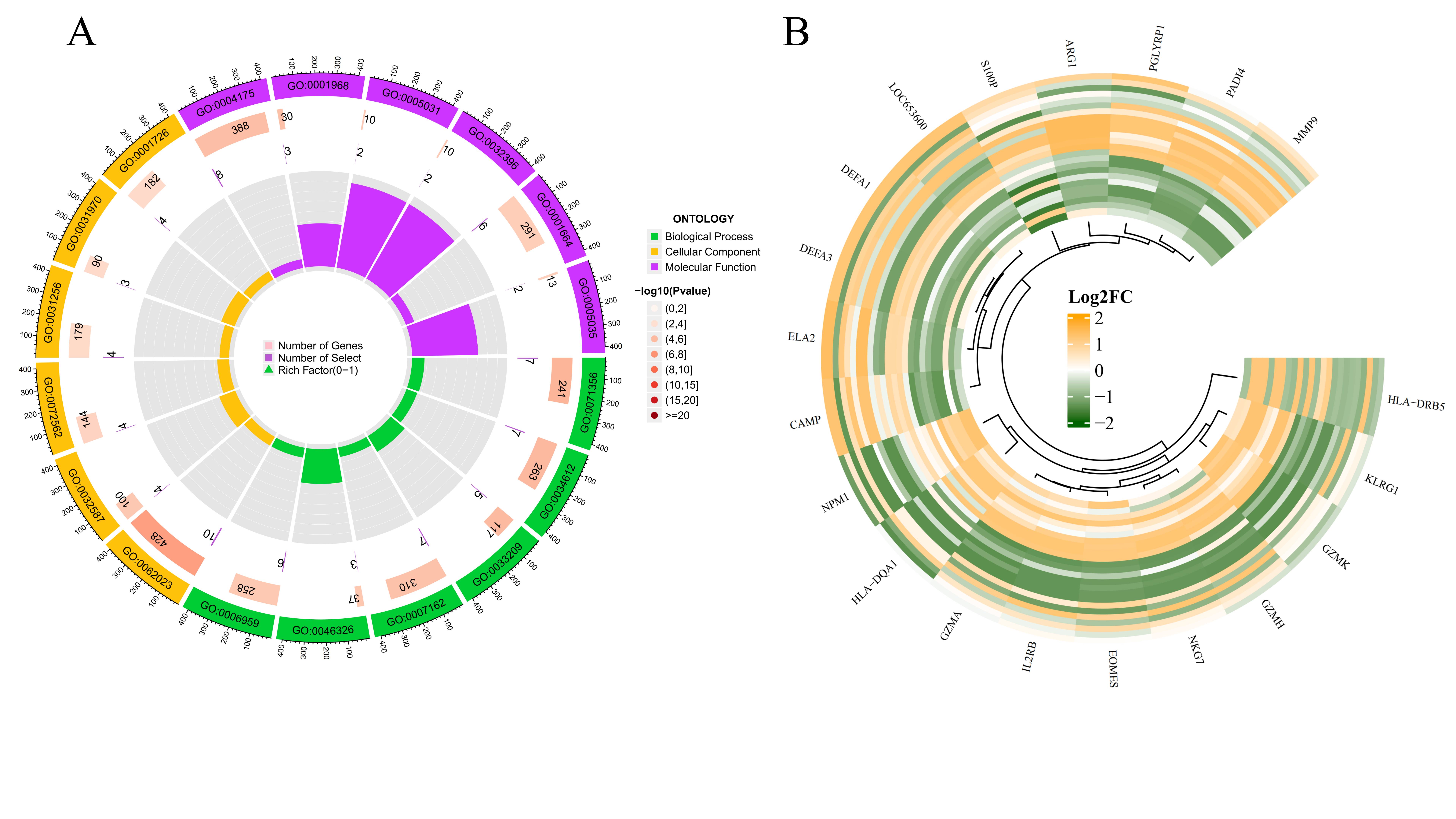


**Figure S1 . Integrated Gene Enrichment and Expression Analysis Visualizations**

1. Focuses on Gene Ontology (GO) enrichment. Different colors distinguish Biological Process (green), Cellular Component (orange), and Molecular Function (purple). The inner circle details the number of genes, selected genes, and Rich Factor, while the outer circle shows GO terms and -log10(P value), highlighting enriched biological aspects. (B) Displays Log2FC values for genes. Colors represent various Log2FC ranges, with each arc for a gene indicating expression change magnitude. -log10(P value) is also depicted, helping to assess significance. Together, they aid in comprehensively understanding gene enrichment and expression changes.


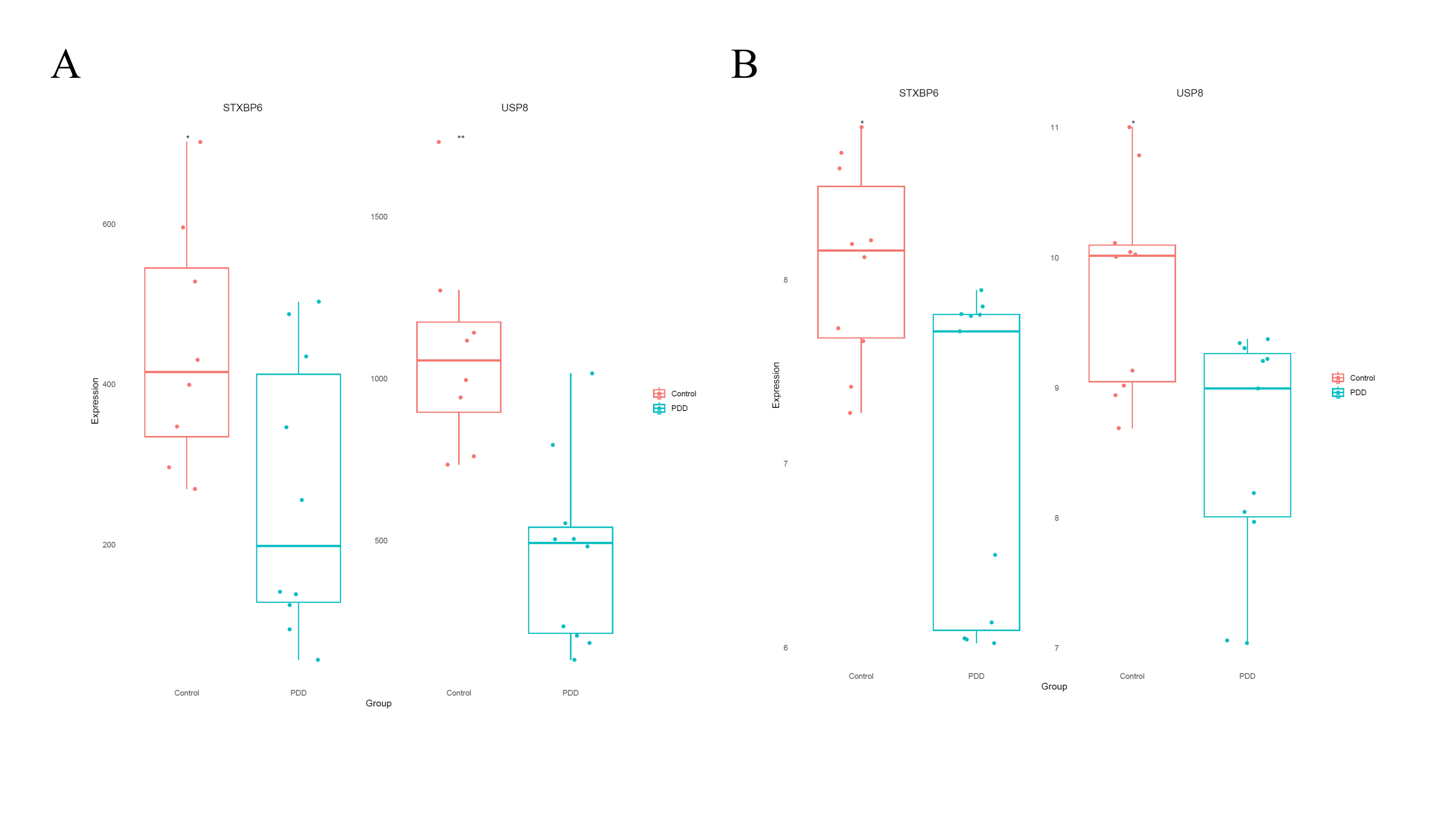


**Figure S2 .Comparative Gene Expression Analysis of STXBP6 and USP8 between Control and PDD Groups**

Figure (A) and (B) box plots display the expression levels of STXBP6 and USP8 genes in the Control and PDD (presumably a disease - related group) groups. Each box represents the distribution of gene expression values, with the central line indicating the median, the box edges denoting the first and third quartiles, and the whiskers showing the range of data, excluding outliers. Individual data points are also plotted.


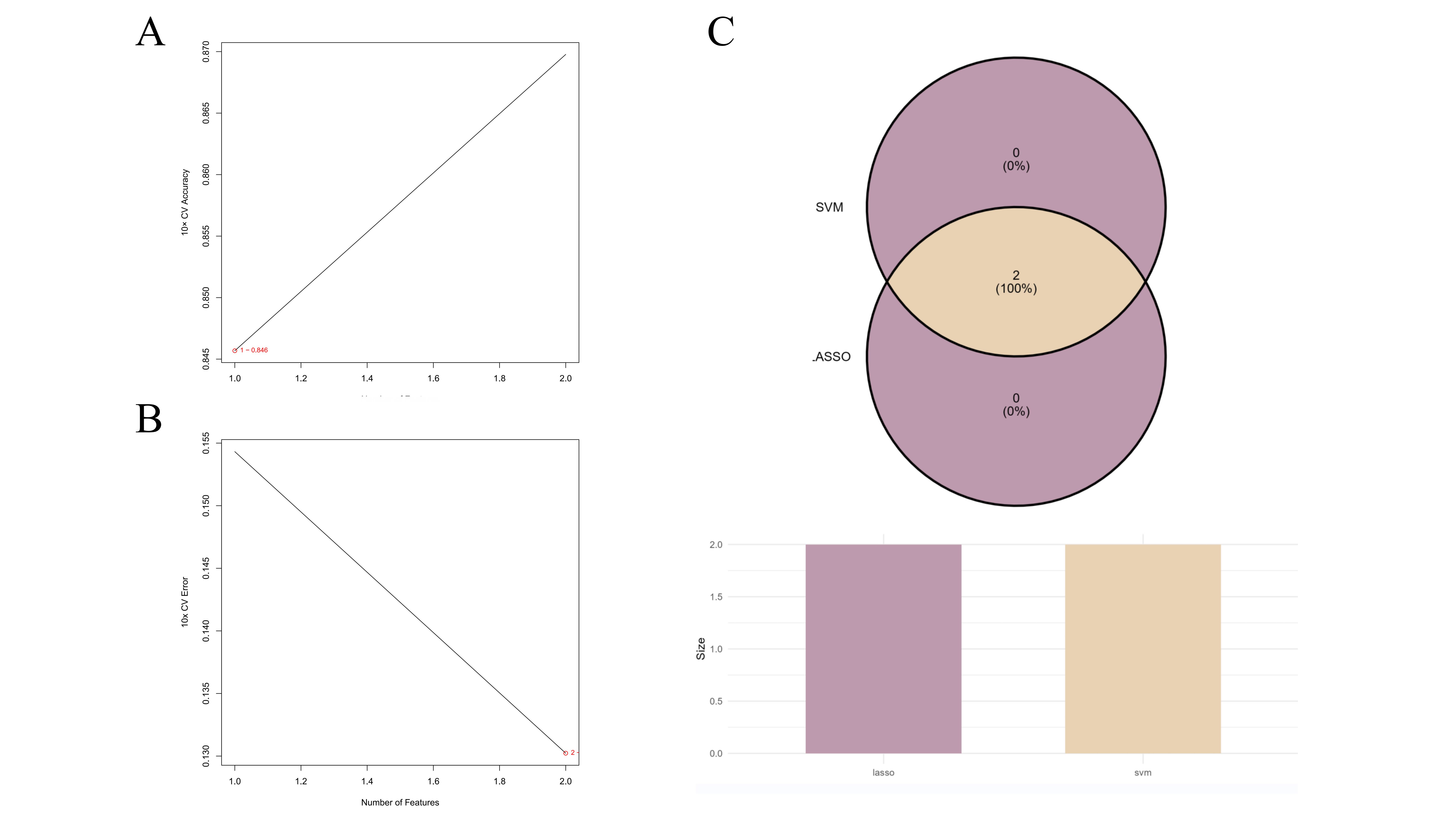


**Figure S3 .Feature-Number Impact on Model Metrics and Algorithm Comparison**

1. Shows a line plot with the x - axis representing the Number of Features and the y-axis showing 10- cv Accuracy. It likely illustrates how the accuracy of a 10-fold cross-validation changes as the number of features varies. (B) Has the same x-axis but the y - axis represents 10-cv Error, depicting the error rate in a 10-fold cross-validation with different numbers of features. These two plots help in optimizing feature selection for a model. (C)Consists of a Venn diagram and a bar plot. The Venn diagram compares SVM and LASSO algorithms, showing that there are 2 common elements between them, accounting for 100% of the overlapping part. The bar plot below likely shows the size or some related metric of the two algorithms, providing insights into their comparison in the analysis context.


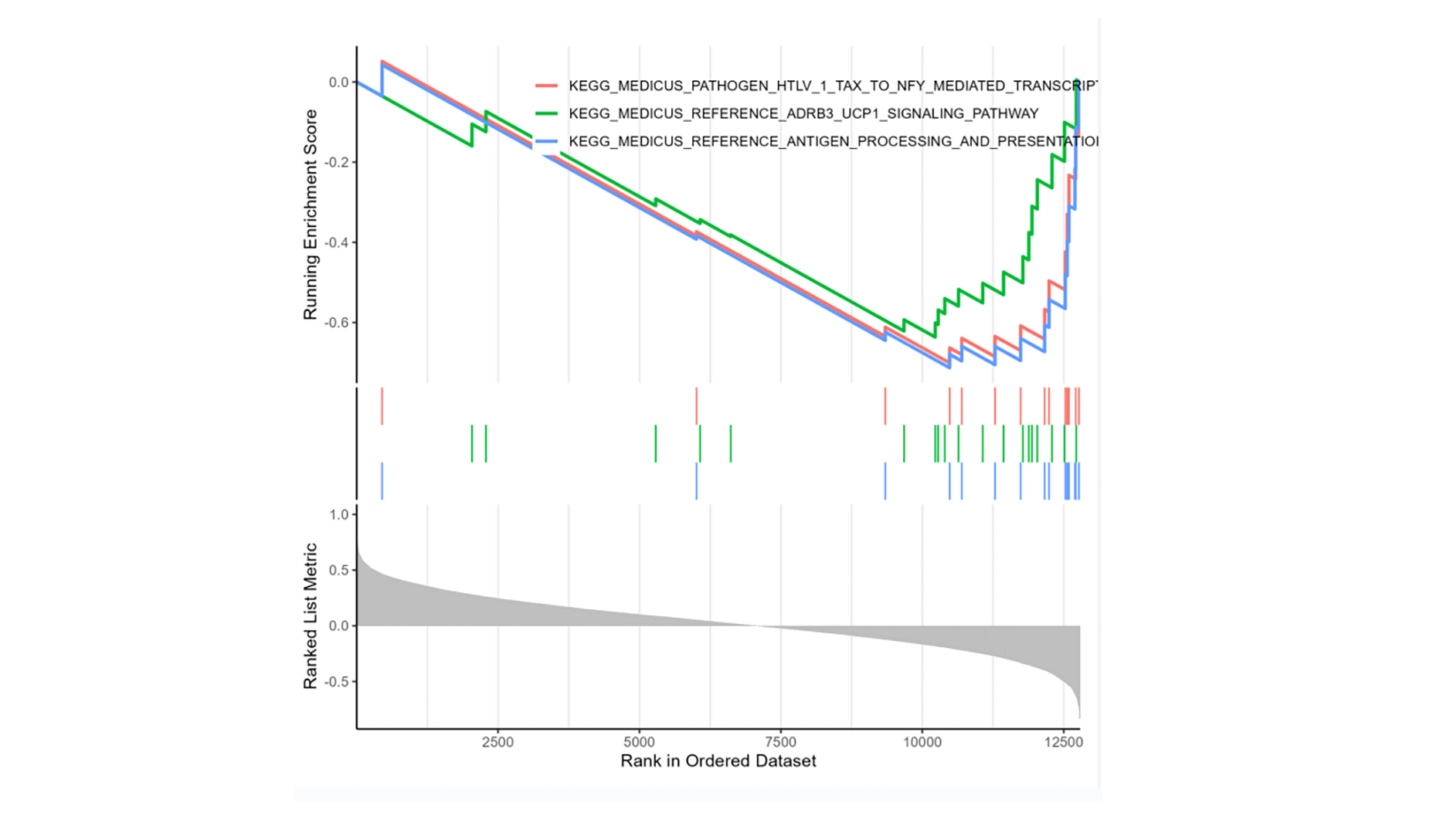


**Figure S4 .Temporal Enrichment Patterns of Key KEGG Pathways**

This plot showcases the Running Enrichment Score trends for three distinct KEGG pathways over the Rank in Ordered Dataset. The pathways, namely"KEGG_MEDICUS_PATHOGEN_HTLV_1_TAX _TO_NFY_MEDIATED_TRANSCRIP","KEGG_MEDICUS_REFERENCE_ADRB3_UCP1_SIGNALING_PATHWAY",and"KEGG_MEDICUS_REFERENCE_ANTIGEN_PROCESSING_AND_PRESENTATION", are each represented by a different - colored line. The x - axis indicates the position in the ordered dataset, while the y - axis depicts the Running Enrichment Score, which gauges the enrichment level of genes in these pathways. The Ranked List Metric below provides additional context on gene ranking. This figure helps in assessing the significance and progression of enrichment for these key biological pathways.


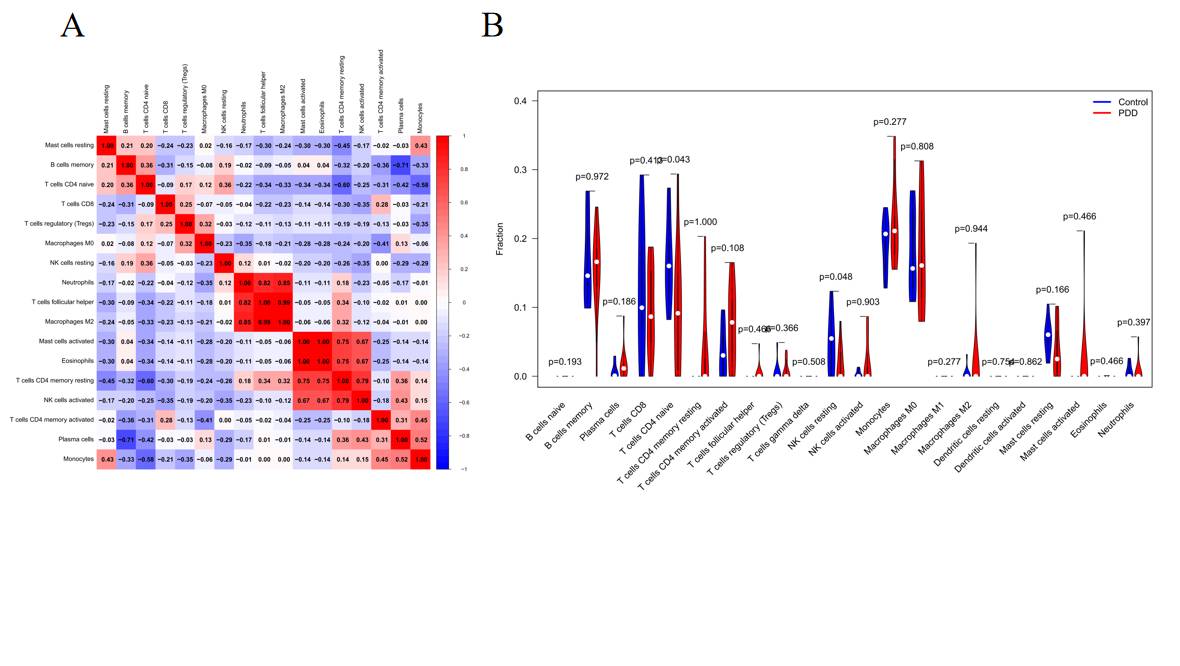


**Figure S5 .Heatmap of Immune Cell Parameter Differences and Violin Plots Comparing Immune Cell Distributions in Control and PDD Groups**

(A)Heatmap depicting the differential values (possibly gene expression levels or functional indices) across a diverse range of immune cell types. Each cell type is listed on the vertical axis, with the horizontal axis likely representing different experimental conditions or samples. The color scale indicates the magnitude of the values, where red signifies higher positive values and blue represents lower negative values. This visualization aids in identifying patterns of up-or down-regulation among various cell types. (B)Violin plots comparing the fractional distributions of immune cell types between the Control and PDD cohorts. Each violin plot corresponds to a particular immune cell type, with the blue distribution representing the Control group and the red one representing the PDD group. The plots illustrate the spread and central tendency of the data, while the p-values displayed above each pair of violin plots indicate the statistical significance of the differences in distributions between the two groups, helping to assess whether observed differences are likely due to chance.


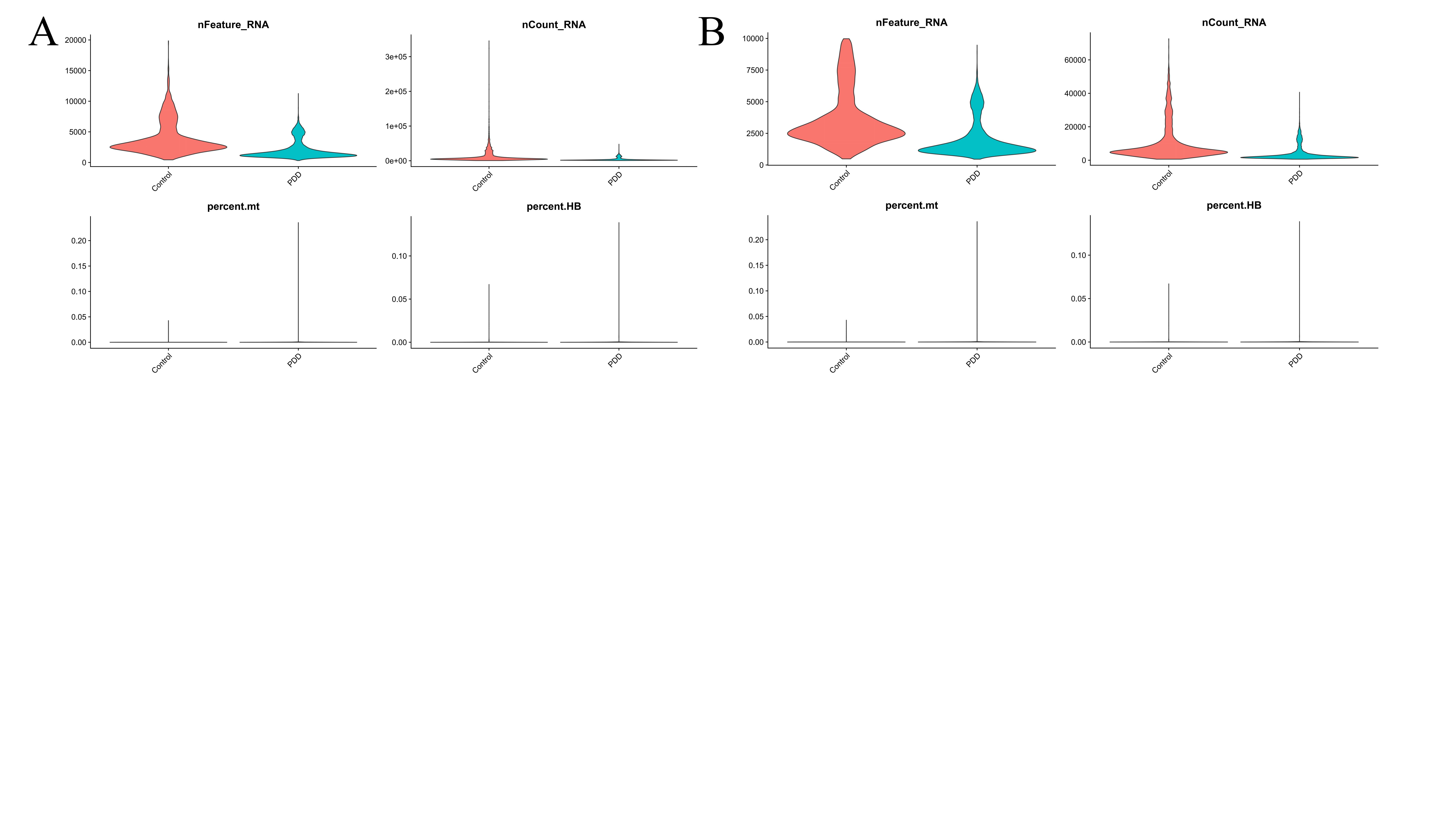


**Figure S6 .Distribution of nFeature_RNA, nCount_RNA, percent.mt, and percent.HB before and after QC**

(A)Distribution of nFeature_RNA, nCount_RNA, percent.mt, percent.HB before QC. (B) Distribution of nFeature_RNA, nCount_RNA, percent.mt, percent.HB after QC.

**
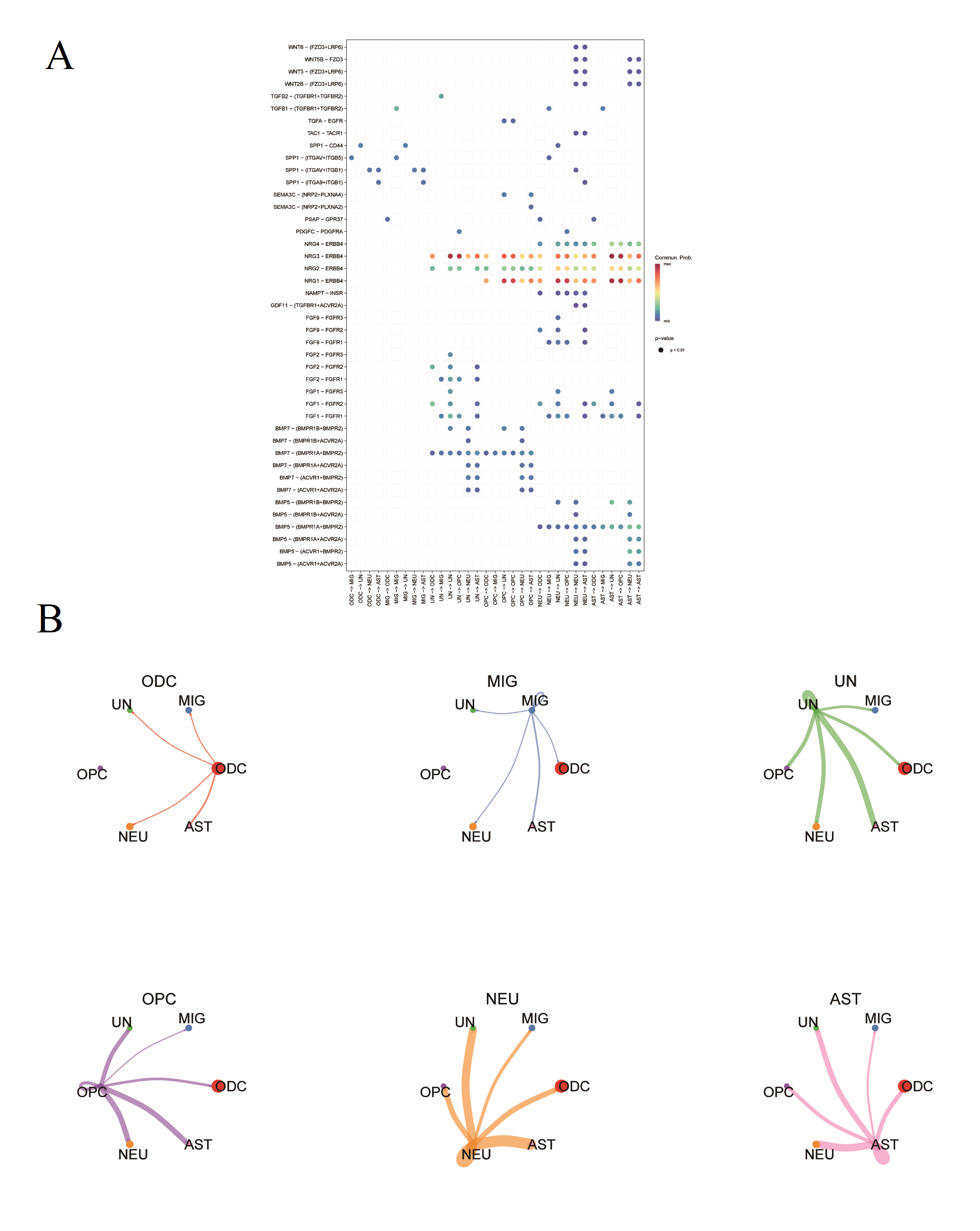
**

**Figure S7.**Visualizing Cell-Cell Interaction Patterns****

**(A)A scatter plot visualizing interactions. Dots are interaction pairs, and the color-code on the right indicates categories or significance. It helps analyze interaction patterns.(B)Network diagrams for cell identities like ODC, MIG. Lines show cell-cell interactions. Color and thickness differences may represent strength or type, aiding understanding of cell communication dynamics.**

**
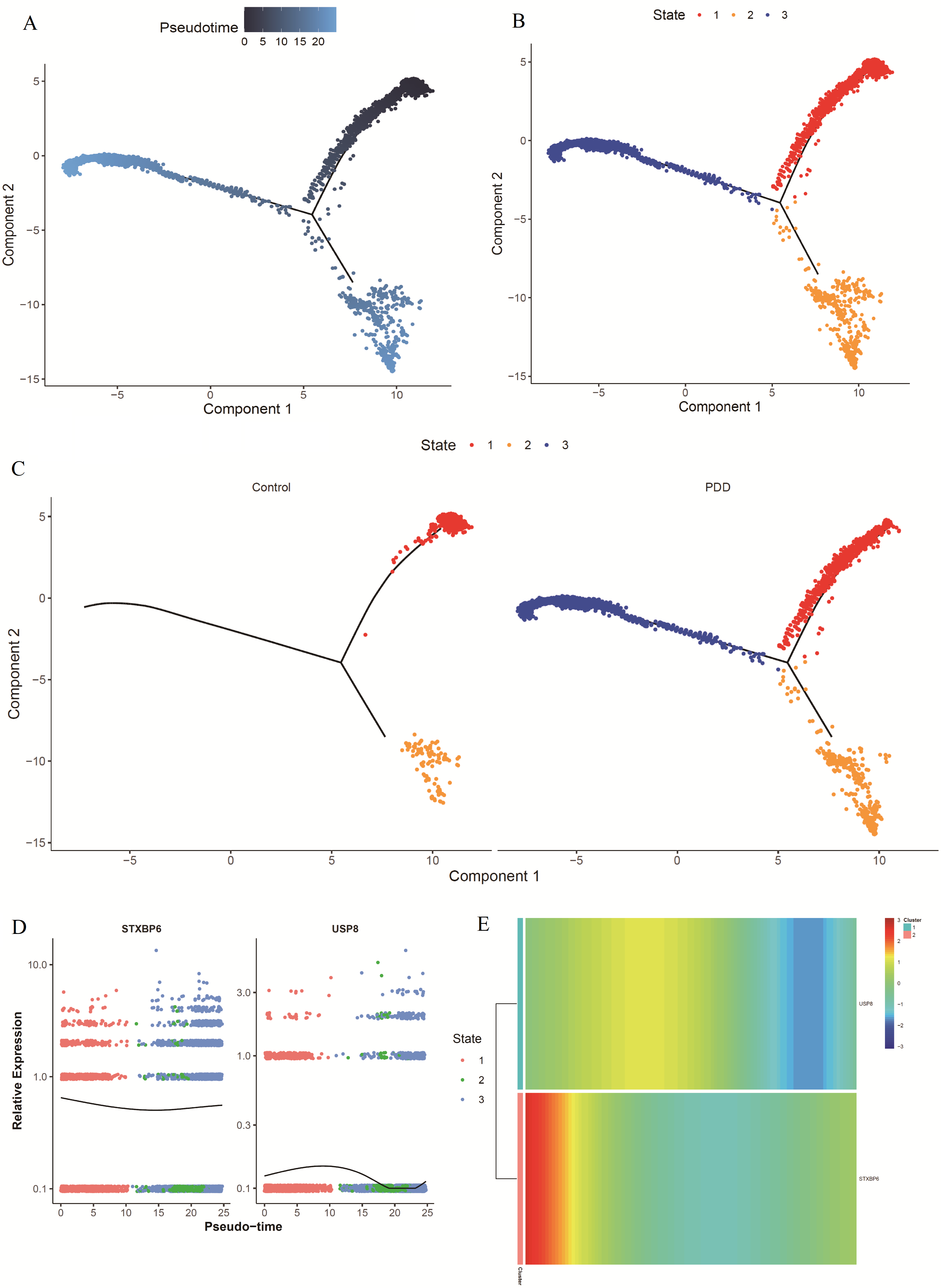
Figure S8 . Pseudotime analysis of ODC cells.** (A) Pseudotime locus map. (B) Locus map of different cell subpopulations. (C) Different differentiation locus stages. (D) Expression of prognostic genes in different temporal stages. (E) Dynamic heat map of prognostic genes.


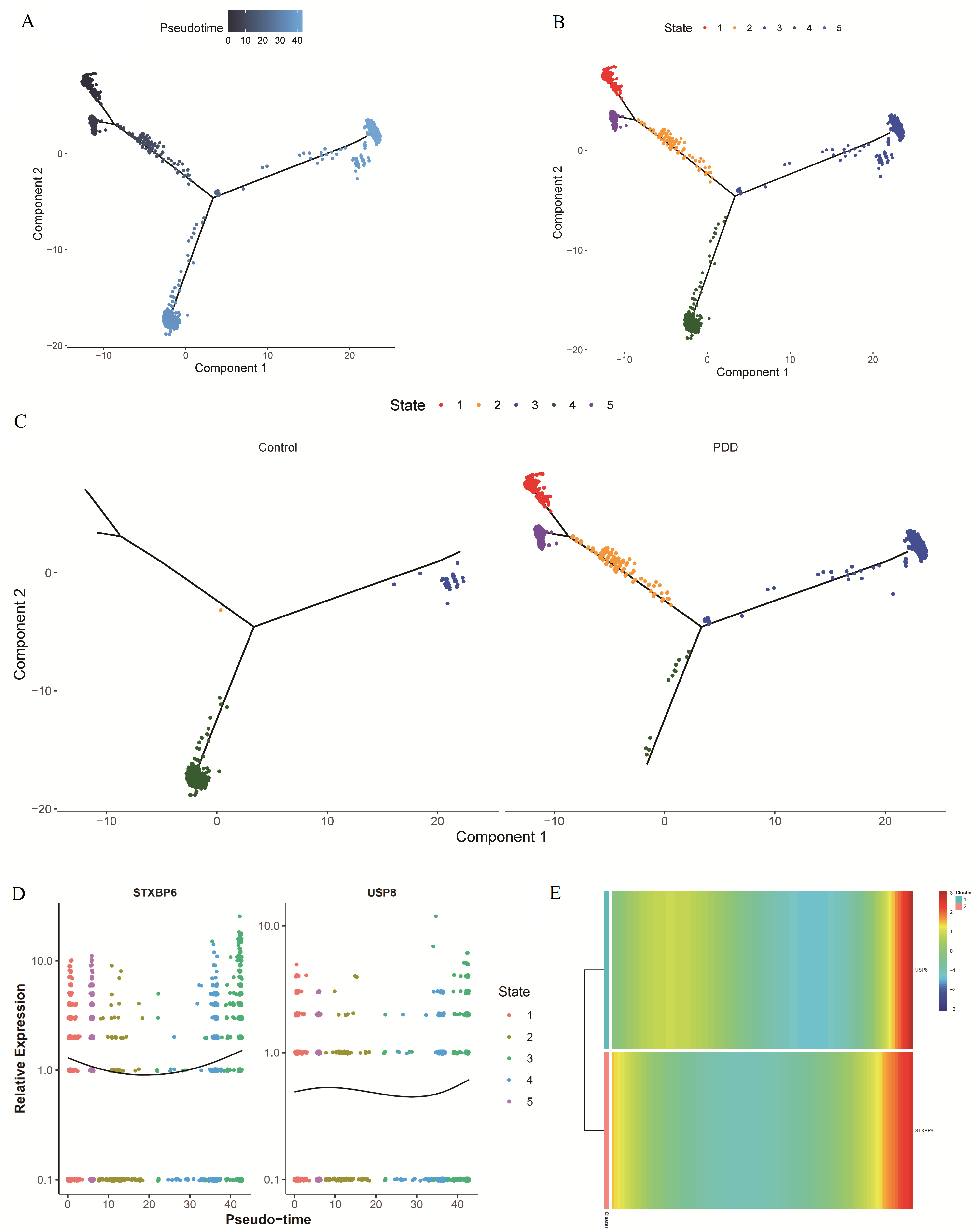


**Figure S9 . Pseudotime analysis of NEU cells.** (A) Pseudotime locus map. (B) Locus map of different cell subpopulations. (C) Different differentiation locus stages. (D) Expression of prognostic genes in different temporal stages. (E) Dynamic heat map of prognostic genes.


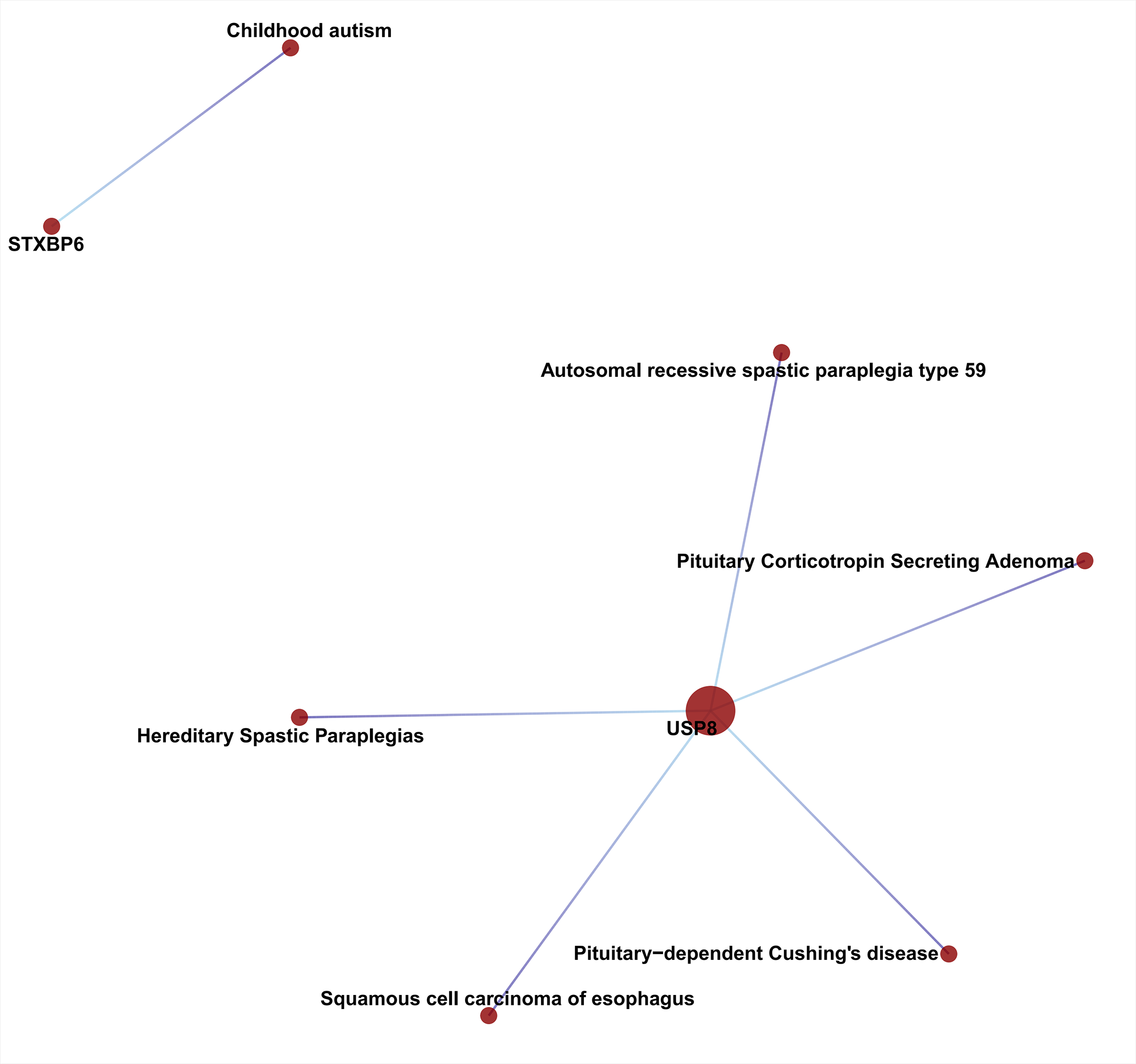


**Figure S10 . Disease-Gene Associations Involving USP8 and STXBP6**

This is a network-style diagram showing the associations between genes (STXBP6 and USP8) and various diseases. USP8, represented by a larger node, is linked to multiple disorders including Autosomal recessive spastic paraplegia type 59, Pituitary Corticotropin Secreting Adenoma, Hereditary Spastic Paraplegias, Pituitary-dependent Cushing's disease, and Squamous cell carcinoma of esophagus. STXBP6 is associated with Childhood autism. These connections likely indicate genetic links or involvement in the pathogenesis of these diseases.

Table S1:

**Table 1 Heterogeneity test**

| x | method | Q | Q_df | Q_pval |
| --- | --- | --- | --- | --- |
| ADAMTS5 | Inverse variance weighted | 5.985335193 | 5 | 0.307648 |
| ADH1C | Inverse variance weighted | 0.801096087 | 2 | 0.669953 |
| AGT | Inverse variance weighted | 0.977323732 | 4 | 0.913215 |
| AKT2 | Inverse variance weighted | 0.818607476 | 6 | 0.991565 |
| ANXA6 | Inverse variance weighted | 2.464222316 | 3 | 0.481792 |
| ARF4 | Inverse variance weighted | 2.702162186 | 7 | 0.911121 |
| ARL1 | Inverse variance weighted | 1.179060961 | 5 | 0.94687 |
| C1QC | Inverse variance weighted | 13.73872534 | 15 | 0.54543 |
| C5orf46 | Inverse variance weighted | 2.72725367 | 6 | 0.842218 |
| CACNA2D3 | Inverse variance weighted | 5.152997042 | 14 | 0.983567 |
| CADM2 | Inverse variance weighted | 1.844561755 | 3 | 0.605286 |
| CCL22 | Inverse variance weighted | 12.48744054 | 15 | 0.641822 |
| CHCHD10 | Inverse variance weighted | 0.489019332 | 2 | 0.783088 |
| CHL1 | Inverse variance weighted | 5.173908236 | 10 | 0.879262 |
| COTL1 | Inverse variance weighted | 1.949566734 | 4 | 0.745035 |
| CRELD1 | Inverse variance weighted | 7.386000352 | 10 | 0.68857 |
| CSAG1 | Inverse variance weighted | 1.837819696 | 5 | 0.8711 |
| CTRC | Inverse variance weighted | 3.610848628 | 4 | 0.461225 |
| CXCL12 | Inverse variance weighted | 5.227171194 | 12 | 0.949958 |
| DCUN1D5 | Inverse variance weighted | 15.74167531 | 12 | 0.203356 |

*Note: Only the first 20 genes are shown.*

Table S2:

**Table 2 Horizontal Multiple Validity Test**

| x | egger_intercept | se | pval |
| --- | --- | --- | --- |
| ADAMTS5 | -0.04685 | 0.082203 | 0.599192 |
| ADH1C | 0.146331 | 0.203186 | 0.602658 |
| AGT | -0.03895 | 0.087478 | 0.68633 |
| AKT2 | 0.041064 | 0.142367 | 0.784589 |
| ANXA6 | 0.213608 | 0.184454 | 0.366444 |
| ARF4 | 0.042265 | 0.106047 | 0.704023 |
| ARL1 | 0.010588 | 0.090258 | 0.912267 |
| C1QC | 0.00614 | 0.060607 | 0.920745 |
| C5orf46 | 0.111031 | 0.119288 | 0.39469 |
| CACNA2D3 | -0.05012 | 0.05244 | 0.356593 |
| CADM2 | -0.08839 | 0.174905 | 0.663505 |
| CCL22 | -0.05585 | 0.052069 | 0.301565 |
| CHCHD10 | -0.08806 | 0.397164 | 0.861088 |
| CHL1 | -0.03928 | 0.065777 | 0.565117 |
| COTL1 | 0.119196 | 0.109465 | 0.355854 |
| CRELD1 | -0.06416 | 0.089279 | 0.490576 |
| CSAG1 | 0.116602 | 0.102532 | 0.318946 |
| CTRC | 0.250925 | 0.155878 | 0.205831 |
| CXCL12 | -0.0559 | 0.066497 | 0.418433 |
| DCUN1D5 | 0.062233 | 0.061126 | 0.3305 |

*Note: Only the first 20 genes are shown.*

Table S3:

**Table 3 Steiger directional analysis**

| X | SNP | steiger_dir | steiger_pval |
| --- | --- | --- | --- |
| ADAMTS5 | rs10922098 | TRUE | 5.81E-08 |
| ADH1C | rs283415 | TRUE | 3.17E-15 |
| AGT | rs139974673 | TRUE | 5.96E-05 |
| AKT2 | rs1042303 | TRUE | 8.00E-07 |
| ANXA6 | rs10822155 | TRUE | 2.69E-11 |
| ARF4 | rs111696008 | TRUE | 0.002388895 |
| ARL1 | rs10822155 | TRUE | 2.95E-05 |
| C1QC | rs10849546 | TRUE | 2.85E-05 |
| C5orf46 | rs35340377 | TRUE | 0.00028421 |
| CACNA2D3 | rs11158602 | TRUE | 0.000143498 |
| CADM2 | rs1260326 | TRUE | 0.005363497 |
| CCL22 | rs10886437 | TRUE | 1.86E-06 |
| CHCHD10 | rs1355538 | TRUE | 9.79E-05 |
| CHL1 | rs10490842 | TRUE | 0.00016257 |
| COTL1 | rs1619994 | TRUE | 4.86E-08 |
| CRELD1 | rs10124390 | TRUE | 0.008159051 |
| CSAG1 | rs10171839 | TRUE | 1.02E-85 |
| CTRC | rs11574452 | TRUE | 0.003438492 |
| CXCL12 | rs10123570 | TRUE | 0.00110847 |
| DCUN1D5 | rs10420758 | TRUE | 0.000492739 |

*Note: Only the first 20 genes are shown.*
